# Graded optogenetic activation of the auditory pathway for hearing restoration

**DOI:** 10.1016/j.brs.2023.01.1671

**Published:** 2023

**Authors:** Artur Mittring, Tobias Moser, Antoine Tarquin Huet

**Affiliations:** a Auditory Circuit Lab, Institute for Auditory Neuroscience and InnerEarLab, University Medical Center Göttingen, Göttingen, Germany; b Institute for Auditory Neuroscience and InnerEarLab, University Medical Center Göttingen, Göttingen, Germany; c Cluster of Excellence "Multiscale Bioimaging: from Molecular Machines to Networks of Excitable Cells" (MBExC), University of Göttingen, Göttingen, Germany; d Auditory Neuroscience and Optogenetics Laboratory, German Primate Center, Göttingen, Germany; e Auditory Neuroscience and Synaptic Nanophysiology Group, Max Planck Institute for Multidisciplinary Sciences, Göttingen, Germany

**Keywords:** Optogenetics, Optical cochlear implant, Neural network, Neuroprosthetic, Chronos, Auditory nerve, Auditory brainstem, Neural stimulation

## Abstract

Optogenetic control of neural activity enables innovative approaches to improve functional restoration of diseased sensory and motor systems. For clinical translation to succeed, optogenetic stimulation needs to closely match the coding properties of the targeted neuronal population and employ optimally operating emitters. This requires the customization of channelrhodopsins, emitters and coding strategies. Here, we provide a framework to parametrize optogenetic neural control and apply it to the auditory pathway that requires high temporal fidelity of stimulation. We used a viral gene transfer of ultrafast targeting-optimized Chronos into spiral ganglion neurons (SGNs) of the cochlea of mice. We characterized the light-evoked response by *in vivo* recordings from individual SGNs and neurons of the anteroventral cochlear nucleus (AVCN) that detect coincident SGN inputs. Our recordings from single SGNs demonstrated that their spike probability can be graded by adjusting the duration of light pulses at constant intensity, which optimally serves efficient laser diode operation. We identified an effective pulse width of 1.6 ms to maximize encoding in SGNs at the maximal light intensity employed here (∼35 mW). Alternatively, SGNs were activated at lower energy thresholds using short light pulses (<1 ms). An upper boundary of optical stimulation rates was identified at 316 Hz, inducing a robust spike rate adaptation that required a few tens of milliseconds to recover. We developed a semi-stochastic stimulation paradigm to rapidly (within minutes) estimate the input/output function from light to SGN firing and approximate the time constant of neuronal integration in the AVCN. By that, our data pave the way to design the sound coding strategies of future optical cochlear implants.

## Highlights

• We demonstrate that spike probability of the spiral ganglion neurons (SGN) and anteroventral cochlear nucleus neurons can be gradually “dialed” by adjusting the width of light pulses like what can be achieved with acoustic clicks of different sound pressure levels. Varying duration rather than light intensity is an important objective as laser diodes operate most efficiently with large driver currents and short pulses.
• Under our experimental condition (light intensity ∼35 mW), we identified an optimal pulse width of 1.6 ms for maximizing information coding in SGNs.
• The lowest energy requirement to activate SGNs was achieved using short light pulses (<1 ms).
• We identified a spike failure induced during high rate optical stimulation (>300 Hz) that likely was due to depolarization block and required a few tens of milliseconds to recover. This is another important constraint that future optogenetic coding strategies need to consider.
• Our data reveal that the timing of optogenetically evoked SGN spikes varied little across iterations for a given neuron but substantially within an SGN population, reminiscent of what is observed acoustically. This insight indicates that optogenetic cochlear implants will likely overcome the problem of overly synchronized activity in the auditory nerve faced by current state of the art electrical cochlear implants.
• We established a new framework to rapidly determine the input/output function from light to firing of the spiral ganglion neurons using a semi-stochastic stimulus. This stimulus allowed us to approximate the time constant of neuronal integration in the neurons of the anteroventral cochlear nucleus, which recode the firing transmitted from the spiral ganglion neurons.

## 1 Introduction

Optogenetics has revolutionized the life sciences by enabling genetically, anatomically and temporally precise interrogations of organ functions, in particular of the brain and its neural circuits (for review, [1]). This technique also unlocked new perspectives for restoring the function in diseased sensory systems by bypassing dysfunctional or lost receptor cells via direct control of the activity of afferent neurons (for review, [2,3]). Cellular specificity and spatial confinement of optogenetic stimulation promise an improvement of functional restoration beyond what is amenable to electrical stimulation currently used in cochlear and retinal implants. The clinical feasibility of this approach was recently proven by the first-in-human trial allowing partial vision restoration in a blind patient [4]. Similarly, optogenetics is an attractive alternative to electrical stimulation for restoring motor function or controlling chronic pain [[5], [6], [7]].

Applying optogenetics to the first neurons of the auditory pathway, the spiral ganglion neurons (SGN), enables new opportunities for improving strategies for hearing restoration [2,3]. We recently demonstrated a major gain in spectral selectivity for optogenetic stimulation of the SGNs over the conventional electric stimulation used by electrical cochlear implants (eCI, [[8], [9], [10], [11], [12]]). This promises that future optical cochlear implants (oCI) will operate with a greater number of independently stimulating channels, which expectedly convey more natural sound perception and improve speech understanding in the presence of background noise as well as music appreciation [[2], [3]]. However, improving the spectral code with optical stimulation should not trade in poor temporal coding e.g. due to slow gating of channelrhodopsins (ChR). Hence, efforts have been undertaken to speed up optogenetic control of SGNs by ultrafast ChRs [[13], [14], [15]].

The outlined case of optogenetic hearing restoration exemplifies that establishing optimal optogenetic control of neural activity in a given circuit is far from trivial. It requires a good tuning of ChR membrane expression and matching of the biophysical opsin properties [[13], [14], [15], [16]] to the neuron's excitability [17], and the identification of optimal simulation parameters, also regarding the operation of the chosen emitters. For a neuronal population of interest, this typically necessitates the empirical measurement of the input/output function from light to spike in different illumination conditions [[16], [17], [18], [19]]. This is also highly relevant to the optogenetic stimulation of the auditory pathway. To date, the expression of various ChRs in SGNs has been achieved (channelrhodopsin-2: [10,20]; CatCh: [9,11,21]; fast and very-fast-Chrimson variants: [13,15]; and Chronos [14,22]). In response to saturating light pulses delivered at a low repetition rate (10–50 light pulse per second, pps), SGNs fire one (CatCh, Chronos and f- and vf-Chrimson) to three (CatCh) action potentials with a sub-millisecond precision (first spike latency jitter ∼ 0.25 ms, [[13], [14], [15],^21^]).

Recordings from cultured hippocampal neurons showed that the maximum repetition rate at which light pulses entrain action potentials is co-determined by the photocurrent amplitude, the closing kinetics of the opsin, and the intrinsic maximum firing rate of the neuron [18,23]. SGNs recorded *in vivo*, show a similar relationship when expressing ChRs with closing time constants ≥3 ms (at body temperature, (CatCh: [21]; f-Chrimson: [15]). Yet, the greatest temporal fidelity that was achieved with faster closing opsins such as Chronos (≤1 ms) and vf-Chrimson (∼1.5 ms) did not enable light-synchronized firing to surpass 200 pps of stimulation [[13], [14]] suggesting an influence of parameters other than the closing kinetics. Another important dimension for bionic encoding is the range of the stimulus intensity which is represented by the neuron's spike rate and spike temporal precision. Recently, we showed a dynamic range of ∼4 or 1 dB in SGNs expressing f- or vf-Chrimson, respectively [13] that indicates that the dynamic range depends on the ChR employed.

To date, multiple unanswered questions remain to be addressed *en route* to the application of optogenetics for hearing restoration. How can we gradually activate neurons at high light irradiance as required for the efficient operation of semiconductor lasers (i.e. using strong and sub-millisecond driver current pulses)? Is there an optimal pulse width for efficient intensity encoding? Does the temporal precision of optically evoked action potentials comply with the integration window of the second-order neurons (i.e. the anteroventral cochlear nucleus, AVCN neurons)?

Here, we addressed these questions by *in vivo* recordings from single SGNs expressing the ultrafast trafficking-optimized Chronos (Chronos-ES/TS, τ_off_ = 0.76 ms at body temperature [14]) in mice. We demonstrated that graded SGN activation (as well as AVCN neuron activation) is achieved by adjusting the width of light pulses. This mimicked what can be achieved with acoustic clicks of different sound pressure levels but constant duration. Under our experimental conditions, we found that with a pulse width of 1.6 ms at maximum light intensity (∼35 mW), SGNs fired most reliably and with high temporal precision. The shorter the light pulses the lower was the energy threshold of activation. Further, we identified that high-rate optical stimulation induced spike failure likely by depolarization block, which took a few tens of milliseconds to recover. Our data revealed that the timing of optogenetically evoked SGN spikes varied little across iterations for a given neuron but substantially within a SGN population. Finally, a novel semi-stochastic stimulus allowed us to rapidly establish the optogenetic SGN input/output function and to generate firing of different statistics for approximating the time constant of neuronal integration in AVCN neurons.

## 2 Results

**Fig. 1:**
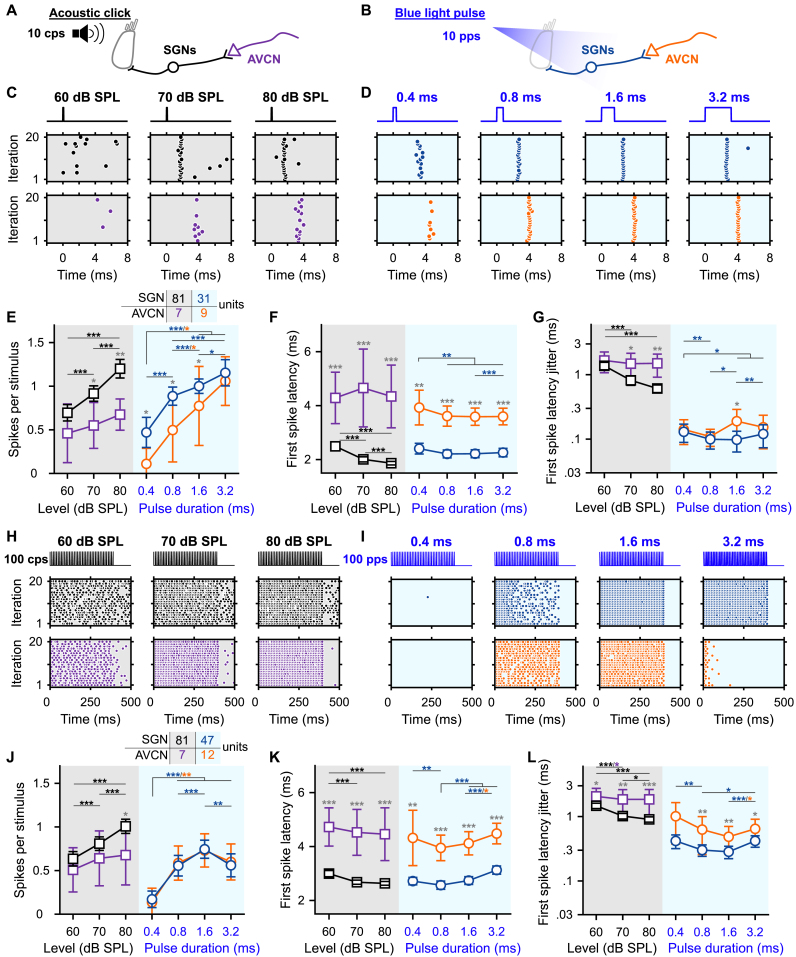
**Gradual optogenetic activation of the first neurons of the auditory pathway with light pulses of increasing durations. A-B.** Schematic of the *in vivo* single unit recordings using sharp micropipettes from spiral ganglion neurons (SGNs, black), optogenetically modified SGNs (blue) or neurons of the anteroventral cochlear nucleus (AVCN, acoustic: purple and optogenetic: orange) in response to acoustic click (A) or optogenetic (B) stimulation delivered to the cochlea from an optical fiber (400 ms train stimulation followed by 100 ms silence/dark, 10–20 trials per condition, all data are paired). **C-D.** Representative raster plots from one SGN (top panel) and one AVCN neuron (bottom panel) in response to single acoustic clicks at various sound pressure levels (C, click trains presented at 10 clicks per second, cps) or single light pulses of different pulse durations (D, ∼35 mW light pulses delivered at 10 pulses per second, pps). Note that acoustic and optogenetic experiments were done from two distinctive cohorts of animals, but SGNs and AVCN neurons were recorded from the same animals respectively. **E-G.** Quantification of the number of spikes per stimulus (E), first spike latency (F) and first spike latency jitter (G) as a function of the click sound pressure level ("click intensity") for the acoustic modality (grey background) or the light pulse duration for the optogenetic modality (blue background). The color code is similar to panels A–D. The number of units per group is presented in the inset of panel E. Data are represented as mean and confidence interval (95%). The effect of the click intensities and light pulse durations was tested by a Wilcoxon signed rank test on paired samples followed by a Bonferroni correction of the *p*-values. The difference between SGNs and AVCN neurons was tested by a two-sample *t*-test or a Wilcoxon rank sum test according to the outcome of Jarque-Bera normality testing (grey symbols; ∗, *P* ≤ .05; ∗∗, *P* ≤ .01; ∗∗∗, *P* ≤ .001). **H-L.** Same as C-G in response to 100 cps acoustic click or 100 pps light pulse trains. Note that the units presented in H–I are the same as in C-D. (For interpretation of the references to color in this figure legend, the reader is referred to the Web version of this article.)

To characterize the encoding of optogenetic cochlear stimulation by the first two neuronal populations of the auditory pathway, we performed stereotactic *in vivo* single unit recordings from SGNs and AVCN neurons in response to acoustic (Fig. 1A) or optogenetic Fig. 1B) stimulations. For optogenetic stimulation, SGNs were transduced by a postnatal AAV injection to express the fast-closing, trafficking-optimized Chronos (Chronos-ES/TS) under the control of the human synapsin promoter [14]. The light was delivered in the cochlea via an optical fiber coupled to a blue laser λ = 473 nm. Out of 55 injected mice, 49 showed an optogenetic activation of the auditory pathway characterized by optogenetically evoked auditory brainstem responses (oABR, Fig. S1. A-C, P_1_–N_1_ amplitude at light intensity (or radiant flux, radiant energy) of 35 mW, 35 μJ = 2.00 ± 0.41 μV, threshold = 12.19 ± 1.59 mW, 12.19 ± 1.59 μJ, average ± 95% confidence interval). We note that, unfortunately, our experimental set-up did not allow a direct estimation of the irradiance at the respective cochlear positions. Instead, we have previously employed Monte Carlo simulations of optical rays emitted from light sources placed at different cochlear positions to estimate the irradiance at the neural soma of the spiral ganglion neurons. Dependent on the light intensity, position and beam profile of the light source, we found maximal irradiances at the neural soma of up to 60 mW/mm^2^ [9,12,21]. Moreover, the neural response also depends on the AAV-mediated ChR expression. Here, across all AAV-injected cochleae, 64.14 ± 5.97% of the SGNs were transduced (Fig. S1). The lowest oABR thresholds were measured for the cochleae with the highest transduction rate (Fig. S1.K, correlation coefficient = −0.44, *P =* .00039). Interestingly, the oABR amplitude was not correlated with the transduction rate (Fig. S1.L).

### 2.1 Graded optogenetic activation of spiral ganglion neurons by light pulses of increasing duration

Laser diodes, which are potential candidates of semiconductor emitters for future optical cochlear implants, show their best efficiency at strong and brief driver currents. This technical requirement imposes that gradual SGN activation is achieved by light pulses of different durations. Gradual neuronal activation can be optogenetically achieved by controlling the number of photons reaching the channelrhodopsins (ChR) expressed at the membrane of neurons. Experimentally, this can be done by varying the light intensity and/or the light pulse duration [[14], [15], [16],^18^,^21^].

First, we assessed the encoding of the optogenetic and acoustic stimulus strength in SGNs. We varied the light pulse duration at a fixed strong light intensity. We compared optogenetic responses to those evoked by brief acoustic stimuli (submillisecond clicks of variable sound pressur levels, refered later as "click intensities", but fixed duration) recorded from non-optogenetically modified SGNs (Fig. 1C-D, top row; optic, *n* = 31 SGNs recorded from 6 mice, maximal laser output stimulation = 35 ± 0.35 mW, 35 ± 0.35 μJ, average ± 95% confidence interval; acoustic, *n* = 81 SGNs recorded from 8 mice). All conditions of stimulus strength (light pulse durations for the optogenetically modified SGNs or acoustical click intensities for the native SGNs) were recorded from the same neuron to enable paired comparison between the different conditions. The number of spikes per stimulus increased with the light pulse duration from 0.47 ± 0.17 at 0.4 ms to 1.15 ± 0.15 at 3.2 ms (*P =* 1.03 × 10^−5^, Wilcoxon signed rank test on paired samples followed by a multi-comparison test); and with the acoustic click intensity (Fig. 1C-E) from 0.69 ± 0.09 at 60 dB SPL_PE_ to 1.2 ± 0.11 at 80 dB SPL_PE_ (*P =* 2.84 × 10^−14^). The optimal representation of one spike per stimulus was achieved by a 1.6 ms light pulse and an acoustic click between 70 and 80 dB SPL. Both, varying light pulse durations and click intensities gradually activated the input of the auditory pathway.

The latency and temporal precision of the first spike elicited per stimulus also showed a strong dependence on the stimulation strength (Fig. 1F-G). Optogenetically, the first spike latency was significantly longer for light pulses of 0.4 ms (2.40 ± 0.20 ms) compared to light pulses of 0.8, 1.6 and 3.2 ms (2.21 ± 0.15 ms, 2.22 ± 0.15 ms and 2.26 ± 0.16 ms respectively, *P* ≤ .0021, Wilcoxon signed rank test on paired samples followed by a Bonferroni correction). The temporal precision was highest in response to light pulses of 1.6 ms (first spike latency jitter = 97.8 ± 34.7 μs) and 0.8 ms (99.4 ± 29.4 μs) compared to 0.4 and 3.2 ms light pulses (129.9 ± 41 μs and 120.3 ± 46.7 μs respectively, *P* ≤ .015, Wilcoxon signed rank test on paired samples followed by a Bonferroni correction). Acoustically, the first spike latency decreased from 2.49 ± 0.17 ms at 60 dB SPL_PE_ to 1.86 ± 0.06 ms at 80 dB SPL_PE_ (*P =* 8.19 × 10^−11^). Likewise, the jitter of the first spike latency decreased from 1.37 ± 0.18 ms at 60 dB SPL_PE_ to 0.61 ± 0.08 ms at 80 dB SPL_PE_ (*P =* 5.51 × 10^−9^). Surprisingly, given the need for cochlear sound processing upstream of SGN spike generation in the auditory pathway, the first spike latency in response to 80 dB SPL_PE_ acoustic click was significantly shorter than all optogenetically tested conditions (*P* ≤ .0019, Kruskal-Wallis test followed by a Tukey's Honestly Significant Difference Procedure). Yet, direct light activation of SGNs showed smaller (one order of magnitude) temporal jitter of the first spike than for acoustic stimulation.

Apart from SGNs, we also recorded from AVCN neurons (see methods section for differentiation between recordings from SGNs and AVCN neurons). We discarded about 30% of the AVCN neurons that encoded the stimulus weakly (<0.3 spikes/stimulus for any of the tested conditions). For all tested conditions, the first spike latency of AVCN neurons was significantly longer than for SGNs reflecting the SGN conduction and synaptic transmission (Fig. 1C, F, *P* ≤ .0033, Jarque-Bera test followed accordingly by a two-sample *t*-test or a Wilcoxon rank sum test). For most tested conditions, the representation of the stimulus in terms of the number of spikes was weaker for AVCN neurons compared to SGNs (0.4, 0.8 and 1.6 ms light pulse: *P* ≤ .0159; 70 and 80 dB SPL_PE_ acoustic clicks: *P* ≤ .0114). Interestingly, this difference decreased for optogenetic stimulation with increasing stimulation duration (strength) from 0.36 at 0.4 ms to 0.09 at 3.2 ms. For acoustic stimulation, in contrast, the difference increased with stimulation strength from 0.24 at 60 dB SPL_PE_ to 0.53 at 80 dB SPL_PE_. This suggests a better recoding of optogenetic stimuli compared to acoustic stimuli by the recorded AVCN neurons.

Next, we investigated if the graded activation of the auditory pathway by various durations of individual light pulses was present also during light pulse trains of high repetition rates (100, 316 and 1000 pps, Fig. 1H-L, Fig. S2). Again, we compared optogenetic stimulation to acoustic stimulation, here at various intensities. At 316 and 1000 pps, the firing of most SGNs fully adapted (i.e. spike rate decreased to 0 spikes/s) within the first few presentations of the light pulses (Fig. S2.C-F). This rapid spike rate adaptation is consistent with our previous recordings of SGN firing mediated by Chronos-ES/TS [14] and vf-Chrimson [13]. Hence, we focused the analysis on 100 pps stimulation trains. While the number of spikes per stimulus was generally lower for light pulse trains, we observed a dependence of SGN firing on stimulus strength similar to what was found for individual light pulses/clicks. Optogenetically the best representation of the light pulse in the SGNs firing was achieved at 1.6 ms (0.74 ± 0.10 spike/stimulus) and decreased for shorter and longer pulse durations (0.4 ms: 0.17 ± 0.09; 0.8 ms: 0.56 ± 0.12; 3.2 ms: 0.56 ± 0.13, *P* ≤ .0012, Wilcoxon signed rank test on paired samples followed by a Bonferroni correction). Spike rate adaptation (i.e. adaptation ratio ≥1, the ratio of the discharge rate between the first 50 ms of the train and the first 400 ms) was evident throughout pulse durations. Adaptation was significantly less at 1.6 ms than at 0.8 and 3.2 ms (Fig. S2.B, *P* ≤ .0012, Wilcoxon signed rank test on paired samples followed by a Bonferroni correction). Upon acoustic stimulation, the number of spikes per stimulus increased approximately linearly with the click intensity from 0.63 ± 0.08 at 60 dB SPL_PE_ to 1.00 ± 0.08 at 80 dB SPL_PE_. No adaptation of the firing rate was observed at any tested click intensity (adaptation ratio ∼ 1, Fig. S2.B). The timing and temporal precision of the first spike were differently affected by the stimulation strength between the 2 modalities. Optogenetically, the shortest first spike latency was measured for light pulses of 0.8 ms (Fig. 1K, 2.56 ± 0.15 ms, *P* ≤ .0093) and the precision was the highest for light pulses of 0.8 and 1.6 ms (Fig. 1.L, first spike latency jitter = 0.30 ± 0.06 ms and 0.28 ± 0.06 ms respectively, *P* ≤ .02 compared to 0.4 and 3.2 ms). Acoustically, however, the first spike latency and first spike latency jitter decreased with the sound pressure level. It is worth mentioning that acoustically, the temporal precision (i.e. the inverse of the spike jitter) was similar for single pulses and 100 pps train stimuli, while optogenetically the precision decreased by a factor of ∼3.

At the AVCN level, the number of spikes per light pulse was comparable to what was observed for SGNs (Fig. 1J). In contrast, the number of spikes per acoustic click was reduced in the AVCN neurons compared to the SGNs (80 dB SPL_PE_, *P =* .0257). For all modalities and conditions, the first spike precision was lower for AVCN neurons than for SGNs (Fig. 1.L, *P* ≤ .0268) and, in general, was less sensitive to the stimulation strength.

**Fig. 2:**
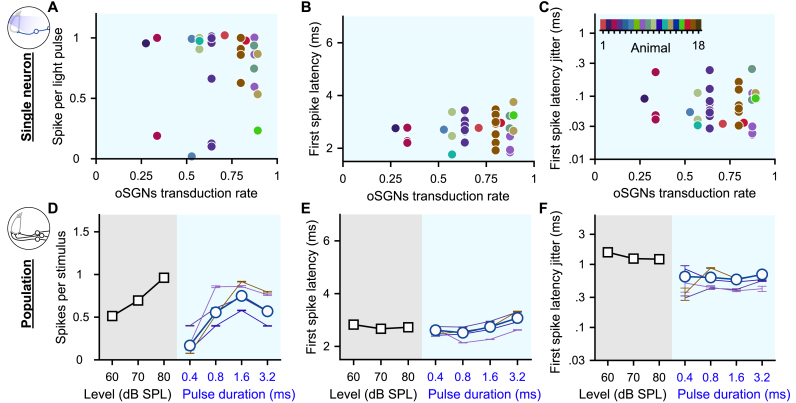
**Heterogeneous optogenetic activation of the SGNs and artificial population response. A-C.** Quantification of the spikes per light pulse (A), first spike latency (B) and first spike latency jitter (C) as a function of the average SGN transduction rate. Measurements were done in response to 100 pps light pulse trains of 1.6 ms (*n* = 35 SGNs). A color code was used per animal. No correlation was found (correlation coefficient test). **D-F.** Quantification of the number of spikes per stimulus (D), first spike latency (E) and first spike latency jitter (F) computed across SGNs recorded from a single animal (≥4 SGNs, thin colored line, same color code as in A-C) and all recorded SGNs (grey background, 100 cps acoustic click train: *n* = 20 SGNs with the best frequency within the 16 kHz octave band; blue background, optogenetics, 100 pps light pulse trais: *n* = 47 SGNs). (For interpretation of the references to color in this figure legend, the reader is referred to the Web version of this article.)

The encoding of light pulses varied widely across SGNs, even when recorded from the same animal and it seemed independent of the transduction rate measured for the animal's cochleae (Fig. 2A-C, *n* = 35 SGNs from 16 mice, 100 pps light pulse train of 1.6 ms, no correlation was found, correlation coefficient function in MatLab). The origin of this heterogeneity is currently unknown, but possible mechanisms include *i)* heterogeneous ChR expression of the SGNs, *ii)* different distances of the SGNs to the light source, and *iii)* varying neuron excitabilities, resulting from variability in the voltage gated ion channels [[25], [26], [27], [28]]. As a result, AVCN neurons might receive excitatory inputs that are less synchronous than originally quantified from individual optogenetically driven SGNs. To approximate this effect, we computationally simulated a population of SGNs from our single SGN recordings and measured the statistics of the spike trains across SGNs per stimulus presentation (Fig. 2D-F, see Methods for details, 50 bootstraps per condition; optogenetics, *n* = 47 SGNs; acoustic, *n* = 20 SGNs with the best frequency within the 16 kHz octave band). As expected, the number of spikes per light pulse (Fig. 2D) and the latency of the first spike (Fig. 2E) quantified at the population level were similar to the averaged results observed for single SGNs across all pulse durations. In contrast, the first spike latency jitter was strikingly higher at the population level and amounted to half of the population spike jitter measured acoustically (Fig. 2F). This last result suggests that the responses to acoustic and optogenetic modalities are not as dissimilar as suggested by the quantification made on single neurons.

Overall, our results showed that varying the duration of high light intensity pulses enables a graded activation of SGNs similarly to what is achieved by changing the intensity of an acoustic click. The best SGN encoding was obtained with 1.6 ms light pulses (at ∼ 35 mW) and those spike trains were characterized by a high spike time precision (compared to acoustic stimulation). In contrast to previous studies using acoustic stimuli in cats and Mongolian gerbils [29,30], no enhancement of the spike rate or temporal precision was observed within the small population of recorded AVCN neurons compared to the SGNs for both acoustic and optogenetic stimulation.

### 2.2 Identifying the optimal pulse duration to encode light pulse amplitudes

Next, we covaried the pulse duration and the light intensity for a more comprehensive analysis of optogenetic encoding. Acoustically, the stimulus intensity is encoded in individual SGNs, with different activation thresholds [[31], [32], [33], [34], [35]], by changes in their firing rate over ∼5–50 dB). Hence, each of them encodes a fraction of the audible dynamic range of ∼120 dB. The response of the SGNs to sound of various intensities is shaped by multiple mechanisms such as the cochlear active micromechanics [36] and the inner hair cell synapses heterogeneity [37,38], which are bypassed in direct SGN stimulation by cochlear implants. Using electric pulses, the dynamic range per SGNs is limited to ∼1 dB (current level) [39]. We recently reported intensity encoding by ChR-expressing SGNs and the measured dynamic ranges were dependent on the used opsin ranging from ∼1 to ∼4 dB [13].

**Fig. 3:**
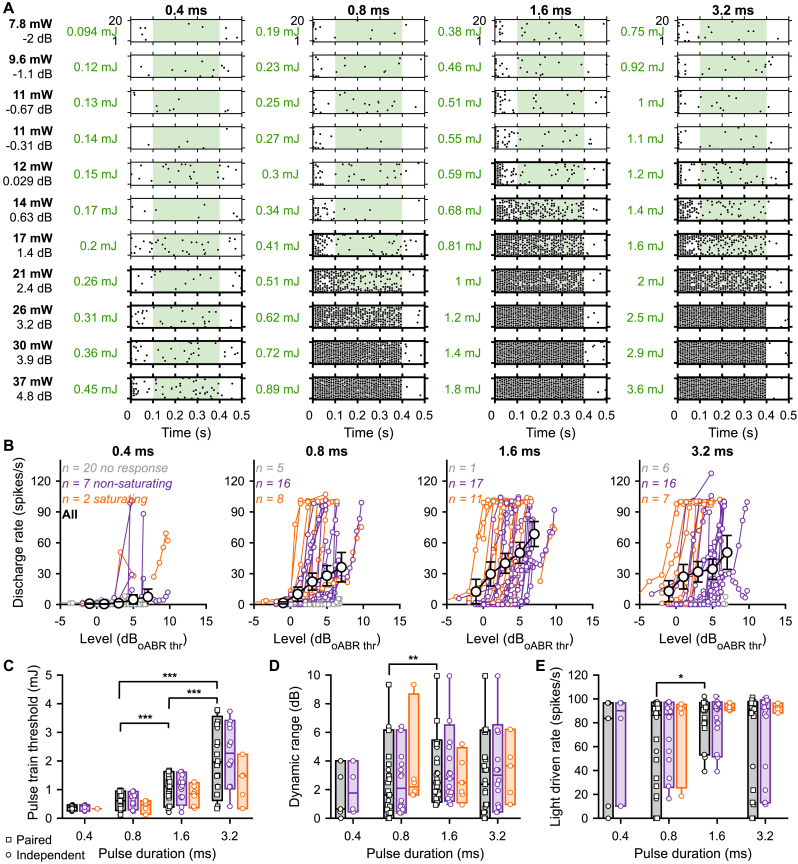
**The best intensity encoding is achieved using 1.6 ms light pulses. A.** Raster plots of a representative SGN evoked by 100 pps light pulse trains (400 ms light, 100 ms dark) at various light intensities and pulse durations. The tested light intensities on the left are expressed in light intensity (or radiant flux in mW), level relative to the oABR threshold (dB_oABR thr._, see Methods) and energy (mJ, calculated within the green window, 30 light pulses). Spike trains were computed on the adapted rate (100–400 ms, green window). Tick raster plot boxes highlight response above the threshold which was determined as the light intensities for which d’ ≥ 1 (see Methods). **B.** Rate level functions of 29 SGNs measured in response to light pulse trains using various light pulse durations. A color code was used to represent non-responding (grey), non-saturating (purple) and saturating (orange) units (see Methods for classification). The average (±95% confidence interval) rate-level function was plotted in black for all pulse durations (intensities values binned between −5 and 8 dB, bin width = 3 dB). Note that the highest light-driven rate was obtained at 1.6 ms. **C-E.** Quantification of the pulse train threshold (C, 30 light pulses), dynamic range (D) and light-driven rate (E) as a function of the light pulse duration. A color code was used to represent all units (black), non-saturating (purple) and saturating (orange) units Box plots show minimum, 25th percentile, median, 75th percentile, and maximum with individual data points overlaid. Circles represent independent data points and squares paired data points at 0.8, 1.6 and 3.2 ms. The effect of light pulse durations was tested at 0.8, 1.6 and 3.2 ms using a Wilcoxon signed rank test on paired samples followed by a Bonferroni correction. Following assessment for normality using a Jarque-Bera test, the difference between non-saturated and saturated units was tested accordingly by one-way analysis of variance or a Kruskal-Wallis test (∗, *P* ≤ .05; ∗∗, *P* ≤ .01; ∗∗∗, *P* ≤ .001). (For interpretation of the references to color in this figure legend, the reader is referred to the Web version of this article.)

To address optogenetic intensity encoding, we recorded the response to 100 pps light pulse trains of 0.4, 0.8, 1.6 and 3.2 ms in a single neuron at as many light intensities as amenable within the time the recording lasted (Fig. 3A, 20 iterations, 400 ms light pulse train, 100 ms dark). Here, we included a total of 29 SGNs from 12 mice for which at least 4 light intensities were presented, and for which the dynamic range was covered from sub-threshold to above the neuron's saturation for at least one of the tested pulse duration (see Methods for details). To facilitate comparison between animals, light intensities were converted to a level relative to the oABR threshold (as introduced in [13], see Methods, dB_rel to oABR thr._). At the threshold, the optogenetic SGN response is characterized by a strong adaptation of firing during the first ∼100 ms [13] following the stimulus onset, therefore the rate-level functions (RLF, Fig. 3B) were built intentionally from the adapted rate (between 100 and 400 ms, green box in Fig. 3A). Additionally, the stimulus energy, between 100 and 400 ms (30 light pulses), was calculated and used for comparing the threshold across SGNs.

RLFs, classified as non-responding, saturating, or non-saturating, could be derived from every tested pulse duration with the highest proportion of responding units observed at 1.6 ms (28 responding units out of 29 recorded ones vs. 9, 23 and 23 responding units at 0.4, 0.8 and 3.2 ms respectively, Fig. 3B). When data permitted, we determined a firing rate-based SGN threshold using d’ statistics [13,40,41]. Expressed as the power relative to the oABR threshold, the single SGN threshold tended to decrease with the pulse duration. Compared to 0.8 ms light pulses (2.97 ± 1.34 dB_rel oABR thr._, average ± 95% confidence interval*, n* = 19 SGNs, Fig. S3), the lowest threshold per SGN was measured at 1.6 ms (1.64 ± 1.24 dB_rel oABR thr._, *P =* .0006, Wilcoxon signed rank test on paired samples followed by a Bonferroni correction). It is worth noting that at 0.8 and 3.2 ms, the RLFs classified as saturated were activated ∼2.5 dB lower than non-saturated RLFs (*P* ≤ .0204, Jarque-Bera test followed accordingly by one-way analysis of variance or a Kruskal-Wallis test). This suggests that all RLFs could have been saturated if a more powerful light emitter would have been used. When the threshold was expressed as the amount of energy required to generate the light pulse trains, the threshold was inversely proportional to the pulse duration (Fig. 3C). The lowest threshold per SGN was measured in response to 0.8 ms light pulse train (30 light pulses) and amounted to 615.03 ± 126.6 μJ (∼20.5 µJ/pulse, 1.6 ms: 921.14 ± 192.4 μJ, *P =* .0004; 3.2 ms: 2034.5 ± 503.6 μJ, *P =* .0004). Next, we measured the dynamic range (i.e. level difference from threshold to 99% of the maximum adapted firing rate for a saturating SGN or to the highest tested level for a non-saturating SGN, *n* = 21 SGNs, Fig. 3D) and the light-driven rate range (i.e. discharge rate difference from threshold to 99% of the maximum driven rate for a saturating SGN or to highest tested level for a non-saturating SGN, *n* = 21 SGNs, Fig. 3E). Again the 1.6 ms stimulations generated the widest dynamic ranges and driven rate (3.28 ± 1.00 dB and 85.41 ± 7.77 spikes/s) compared to 0.8 (2.37 ± 1.15 dB, *P =* .0035; 58.60 ± 18.18 spikes/s, *P =* .0172). Under the limited maximum light intensity (∼35 mW) available, these results suggest that the best intensity encoding in terms of the number of recruited SGNs, thresholds (expressed as intensity), dynamic ranges and light driven rate was achieved using 1.6 ms light pulses.

### 2.3 Recovery from optogenetically-induced masking

In response to ongoing intense sounds (e.g. a few tens of minutes), SGNs continue to fire at a few hundred spikes per second [42]. Optogenetically, most SGNs tonically encode light pulse trains up to a stimulation rate of one to four hundred pulses per second (which depends on the biophysical properties of the opsin) and fully adapt at higher stimulation rates (Fig. S2, [[13], [14], [15],^21^]). How long does it take for SGNs to recover and be responsive again?

**Fig. 4:**
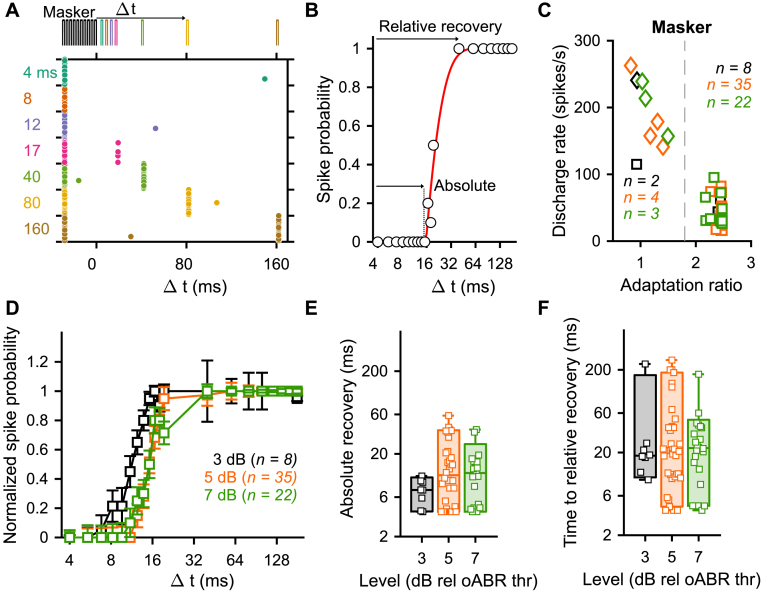
**Recovery from optogenetically induced masking is slow and independent of the stimulation level.** SGN firing was fully adapted using a masker (10 light pulses, 316 pps, 1.6 ms duration) and the recovery of firing was assessed at different time intervals (Δ t) ranging between 4 and 200 ms (20 trials, different laser levels ranging between 3 and 6 dB_rel to oABR threshold_, bin width = 1 dB). **A.** Representative raster plot of a SGN undergoing the forward masking protocol at 4.67 dB_rel oABR thr_. A color code is used to represent the different time intervals. **B.** Recovery curve (i.e. spike probability curve as a function of the time interval) of the unit presented in A. The recovery curve was fitted ($y=\max\left(0,a\times e^{\frac{-\left(k+\Delta t\right)}{b}}\right)$ to define the absolute recovery (i.e. time required to recover any firing, - k) and time to relative recovery (i.e. time required to recover 95% of the spike probability, - k + (3 x b)). **C.** The discharge rate as a function of the adaptation ratio allowed to cluster SGNs as non-adapted (i.e. adaptation ratio ≤1.8 and discharge rate ≥100 spikes/s) and fully adapted. A color code is used for the different levels (D). **D.** The average (±95% confidence interval) recovery curve measured from fully adapted SGNs at 3 (black, *n* = 8), 5 (orange, *n* = 35), 7 (green, *n* = 22) dB_relative to oABR threshold_ (bin width = 2 dB). **E-F.** Quantification of the time to absolute (E) and relative (F) recovery as a function of the light pulse level for the fully adapted SGNs. The same color code is used as in C. Box plots show minimum, 25th percentile, median, 75th percentile, and maximum with individual data points overlaid. No significant difference was observed between the different tested levels (Jarque-Bera test followed by a Kruskal-Wallis test). (For interpretation of the references to color in this figure legend, the reader is referred to the Web version of this article.)

To address this question, we designed a so-called optogenetic forward masking protocol (Fig. 4A, 20 iterations). The stimulus was composed of a masker (10 light pulses of 1.6 ms presented at 316 pps) followed by a single light pulse (1.6 ms) presented at different time intervals (Δ t) ranging from 4 to 180 ms. We primarily employed the maximal light intensity of our experimental system (35 ± 0.35 mW). We, then, repeated the experiment at as many intensities as possible to assess if the recovery was level dependent. We recorded in total 59 SGNs from 11 mice. In about 15% of the recorded SGNs, the firing was not adapted by the masker (Fig. 4C) and therefore recovery could not be assessed. For the adapted SGNs, a normalized spike probability curve was built (Fig. 4B, D) and fitted by a mathematical model to extract the time of absolute (i.e. the time needed to recover any firing, see Methods) and relative recovery (i.e. the time needed to recover 95% of the normalized spike probability). The absolute recovery was level independent and amounted to 7.4 ± 0.67 ms, 16.45 ± 3.88 ms and 12.98 ± 2.64 ms at 3, 5 and 7 dB_rel. to oABR thr._ (Fig. 4E, average ± 95% confidence interval, bin width = 2 dB, Jarque-Bera test followed by a Kruskal-Wallis test). Similarly, the time to relative recovery was level independent and amounted to 36.56 ± 18.98 ms, 44.01 ± 17.60 ms and 27.61 ± 9.41 ms at 3, 5 and 7 dB_rel. to oABR thr_ (Fig. 4F). Recovery time varied starkly and spanned over two orders of magnitude across SGNs, thus revealing another major heterogeneity among them. Acoustically, the time of recovery from a previous stimulus depends on the spontaneous activity of the given SGN: SGNs with a spontaneous activity ≥18 spikes/s recovering the fastest [43]. We did not observe in our data a correlation between the time to relative recovery and the spontaneous activity (Fig. S4.A) or the number of spikes elicited per light pulse (Fig. S4.B). We note that the spontaneous activity of SGNs was highly reduced compared to previous reports under physiological conditions [34]. The spontaneous activity amounted to 0.29 ± 0.11 spikes/s with the highest value at 2.04 spikes/s. We hypothesize that the reduction of spontaneous activity is caused by the loss of the perilymph (e.g. one of the cochlear fluids) following the opening of the cochlea and insertion of the optical fiber.

### 2.4 Semi-stochastic stimulation for rapid estimation of the input/output function from light to neural firing

**Fig. 5:**
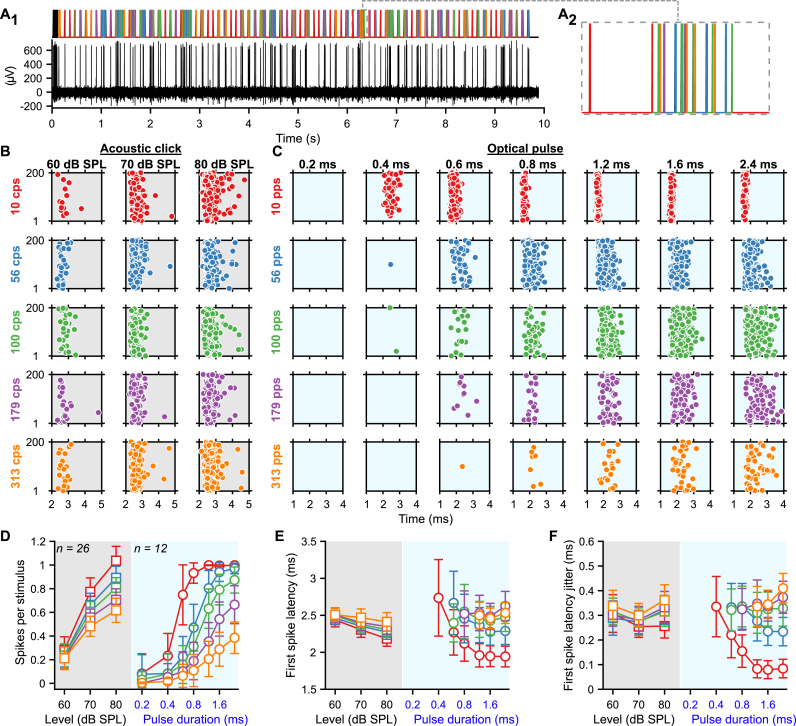
**Input/output function from light to SGN firing can be measured in minutes by a semi-stochastic stimulus.** An optical semi-stochastic stimulus (light intensity = 38 mW) was constructed from 20 presentations of random permutation of repetition rates (10, 56, 100, 179, 313 pps and color-coded in the figure) and light pulse durations (0.2, 0.4, 0.6, 0.8, 1.2, 1.6, 2.4 ms). The stochastic stimulus was preceded by a masker (10 light pulses of 0.8 ms) and followed by 200 ms of dark per trial. It was compared to an acoustic stimulus constructed from 20 presentations of acoustic click trains containing random permutations of repetition rates (10, 56, 100, 179, 313 cps) and presented at 60, 70 and 80 dB SPL_PE_. Additionally, a forward masking protocol was included in the optogenetic stimulus with time intervals of 15 and 80 ms. **A.** Single iteration of the optical stochastic stimulus (A_1_, top and magnification of the stimulus, A_2_) and the response of representative SGNs (A_1_, bottom). **B–C.** Raster plots from representative acoustic (B, grey background) and optically (C, blue background) evoked (o-)SGNs. **D-F.** Quantification of the number of spikes per stimulus (D), first spike latency (E) and first spike latency jitter (F) as a function of the sound pressure level for the acoustic modality (grey background, *n* = 26, best frequency within the 16 kHz octave band) and light pulse duration for the optogenetic modality (blue background, *n* = 13). Data are represented as mean and confidence interval (95%). (For interpretation of the references to color in this figure legend, the reader is referred to the Web version of this article.)

The empirical estimation of the range of optogenetic stimulation properties appropriate for neural encoding is a tedious task. Especially, if all relevant properties have to be tested in a deterministic manner that often results in missing data, which limits the statistical power of the analysis. To overcome this limitation and rapidly measure the input/output function from light to SGN firing, we designed a semi-stochastic stimulus (Fig. 5A) to randomly test 35 combinations of repetition rates (10, 56, 100, 179 and 313 pps) and pulse durations (0.2, 0.4, 0.6, 0.8, 1.2, 1.6, 2.4 ms) by a single round of stimulation. Each combination was randomly tested 10 times per stimulus iteration and the presentation order was randomized for each of the 20 presented iterations, resulting in 200 presentations per combination. Each iteration started with 10 light pulses 2.4 ms presented at 100 pps to adapt the firing and bring the opsin into a desensitized state. Each iteration finished with a 200 ms dark period. Additionally, a forward masking protocol, testing for the time intervals of 15 and 80 ms was included. Consequently, the total duration of the acquisition was 210 s. Recordings were conducted at maximum light intensity (35 ± 0.35 mW). As it was done with the deterministic stimuli before (Fig. 1), the optogenetic stimulus was compared to an equivalent acoustic stimulus, recorded from non-AAV-injected mice, composed of acoustical clicks and presented at various sound levels (60, 70 and 80 dB SPL_PE_).

A total of 13 SGNs were recorded from 2 mice for the optical stimulus and 26 SGNs from 3 mice for the acoustic one. Following reverse correlation and deconvolution (see Methods), raster plots for all tested conditions were built (Fig. 5B-C) and their spike statistics were computed (Fig. 5D-F). As seen for the deterministic stimuli, the number of spikes per stimulus increased with the stimulation strength for both modalities (Fig. 5D). For all light pulse durations or sound intensities, the highest number of spikes per stimulus, shortest latency and highest synchronization were achieved with 10 pps stimulations (Fig. 5D). For the optogenetic modality, higher repetition rates required longer light pulses to evoke the same number of spikes per stimulus and significantly influenced the number and timing of spikes evoked for each light pulse (Fig. S6 for pairwise comparisons of the repetition rates as a function of the pulse duration). Interestingly, reaching the same number of spikes per stimulus with rates >10 pps was always associated with longer latencies and higher jitters (Fig. 5E-F). Thus, the semi-stochastic stimulus allowed to evoke spike trains with similar discharge rates but different spike timing in SGNs.

### 2.5 Heterogeneous optogenetic activation of the SGNs

**Fig. 6:**
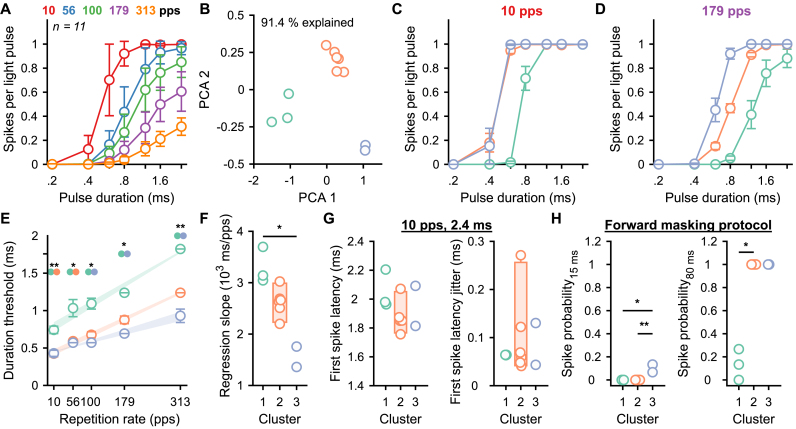
**Heterogeneous responses among SGNs within a single animal. A.** Quantification of the number of spikes per light pulse as a function of the repetition rate and light pulse duration from 11 SGNs measured from the same animal. Data are represented as mean and confidence interval (95%). **B.** The numbers of spikes per light pulse measured at all conditions of repetition rates and light pulse durations were used to perform a principal component analysis (PCA, first 2 components explain 91.4% of the variance) which highlight the presence of 3 SGN clusters (cluster 1, green, *n* = 3; cluster 2, orange, *n* = 6; cluster 3, purple, *n* = 2). **C-D.** Quantification of the number of spikes per light pulse for the 3 SGN clusters as a function of the light pulse duration at 10 (C) and 179 pps (D). Data are represented as the mean and standard error of the mean. **E.** Quantification of the light pulse duration threshold as a function of the repetition rate per cluster. The average linear regression (and standard error of the mean) between threshold and repetition rate is represented by the light fill. The threshold difference between clusters was tested using a Kruskal-Wallis test followed by a Tukey's Honestly Significant Difference Procedure (∗, *P* ≤ .05). **F.** Quantification of the light pulse threshold dependency on the repetition rate (i.e. the slope of the linear regression between the duration threshold and the repetition rate). Box plots show minimum, 25th percentile, median, 75th percentile, and maximum with individual data points overlaid. The difference between clusters was tested using a Kruskal-Wallis test followed by a Tukey's Honestly Significant Difference Procedure (∗, *P* ≤ .05; ∗∗, *P* ≤ .01). **G.** Quantification of the first spike latency (left) and first spike latency jitter (right) measured in response to the condition: repetition rate = 10 pps and light pulse duration = 2.4 ms per cluster. No difference was measured between clusters (Kruskal-Wallis test). **H.** Quantification of the spike probability recovery (forward masking protocol as described in Fig. 3) at 15 ms (left) and 80 ms (right) per cluster. (For interpretation of the references to color in this figure legend, the reader is referred to the Web version of this article.)

We reported heterogeneous optogenetic activation of the SGNs; specifically, the number of spikes evoked per light pulse, the temporal spike precision ranging over 1 order of magnitude (Fig. 2), the activation thresholds ranging over 7 dB, the dynamic range over 9 dB (Fig. 3) and the recovery time from masking ranging over 200 ms (Fig. 4). To investigate the mechanisms underlying this heterogeneity, we capitalized on 11 SGNs that were recorded from the same animal, one after the other in the same condition of optical stimulation and recordings. All neurons were recorded in response to the semi-stochastic stimulus presented above and 2 conditions of forward masking protocol (15 and 80 ms). A principal component analysis on the number of spikes per light pulse for all 35 tested conditions highlighted the presence of 3 clusters (Fig. 6A-B, 91.4% of the variance explained by principal components 1 and 2). Light pulse encoding differed between the three clusters with SGNs from cluster 3 being the most efficient to encode the light pulses and cluster 1 being the worst (Fig. 6C-D). The light pulse duration threshold (i.e. the shortest light pulse required to evoke 0.1 spikes per light pulse) was significantly lower for clusters 2 and 3 compared to cluster 1 and linearly increased with the repetition rate (Fig. 6E, *P* ranging between .0056 and .0278, Kruskal-Wallis test followed by a Tukey's Honestly Significant Difference Procedure). The linear regression slope was significantly lower for cluster 3 compared to cluster 1 (Fig. 6F, *P =* .0138), likely reflecting the differences between the clusters in their dependence of the photocurrent amplitude from the repetition rate.

The latency and jitter of the first spikes evoked by light pulses of 2.4 ms at 10 pps were similar across all clusters. Finally, SGNs from cluster 3 were the fastest to recover from masking (Fig. 6H, spike probability at 15 ms, 0 ± 0, 0 ± 0 and 0.1 ± 0.02 spikes for cluster 1, 2 and 3 respectively, average ± SEM, *p*-values ranging between .0073 and .0193), followed by cluster 2 and 1 (Fig. 6H, spike probability at 80 ms, 0.13 ± 0.07, 1 ± 0 and 1 ± 0 spikes for cluster 1, 2 and 3 respectively, *P* ranging between .008 and .054). Overall, these results demonstrate that SGNs that encode light stimuli the best are also the fastest to recover from the masking. Hence, we postulate that those neurons are the ones with the largest photocurrents (see Discussion).

### 2.6 Enhanced representation of the optogenetic stimulation in AVCN neurons

**Fig. 7:**
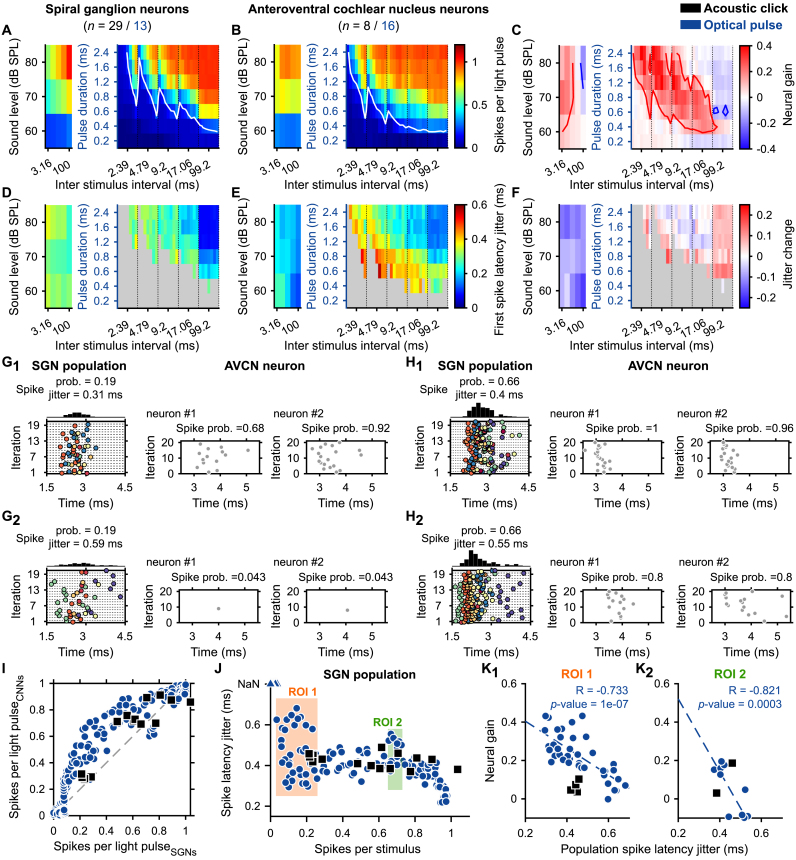
**The neural gain of the AVCN neurons depended on the level of synchronization between SGNs.** The initial 35 conditions of repetition rates and light pulse durations were divided into 245 conditions of inter-stimulus intervals and pulse durations. Note that all conditions were tested randomly for all neurons. **A-B, D-E.** Quantification of the number of spikes per stimulus (A–B) and first spike latency jitter (D–E) for the different tested conditions for SGNs (A, D, acoustic click, black, *n* = 29; light pulse, blue, *n* = 13) and AVCN neurons (B, E, acoustic click, *n* = 8; light pulse, *n* = 16). In A-B, the white line delimits the threshold defined as 0.2 spike/stimulus. **C,F.** Quantification of the neural gain (C) and jitter change (F) between SGNs and AVCN neurons. In C, the red line delimits the area where the neural gain is ≥ 0.1 and the blue line where the neural gain is ≤ 0.1. **G.** (left) Representative raster plots from 2 illumination conditions eliciting the same spike probability (0.19) within the population of recorded SGNs and two different spike jitters (G_1_, 0.31; G_2_, 0.59 ms). Individual SGNs are represented by different colors. (right) Raster plots from two AVCN neurons in response to the same illumination conditions. **H.** Same than G for a spike probability of 0.66 and spike jitters of 0.4 (H_1_) and 0.55 ms (H_2_). **I.** Average number of spikes per light pulse of the AVCN neurons as a function of the number of spikes per light pulse of the SGNs. A blue circle (optogenetic) or a black square (acoustic) corresponds to one of the stimulation conditions tested for all neurons. **J.** First spike latency jitter as a function of the number of spikes per light pulse computed across all SGNs per stimulation condition. Two regions of interest (ROI) were defined as the region where the numbers of spikes per light pulse were in the same range and the variation of the first spike latency jitter was large (ROI 1, 0 ≤ number of spikes per stimulus ≤0.26; ROI 2, 0.65 ≤ number of spikes per stimulus ≤0.73). **K.** Average neural gain as a function of the first spike latency jitter across SGNs in ROI 1 (K_1_) and ROI 2 (K_2_). Correlation coefficients (R and its significance) were measured and regressions were displayed as a dashed line if *P* ≤ .05. (For interpretation of the references to color in this figure legend, the reader is referred to the Web version of this article.)

Above, we reported that the semi-stochastic stimulus allowed the generation of spike trains with different statistics in terms of the number of spikes and the spike timing (latency and jitter, Fig. 5) in the same neuron. Here, we investigated if this property can be used to study the firing from the SGNs to AVCN neurons. First, we observed for all tested repetition rates that the success of triggering a spike with an optogenetic stimulus depended on the success/failure of the preceding light pulse. If the preceding light pulse had a sub-threshold duration, the likelihood of the neuron endocing the following light pulse was high. In contrast, if the previous light pulse had a duration above the threshold, the likelihood to fire upon the following pulse was low. This explains the sawtooth pattern of the response in Fig. 7A-B. Therefore, spike statistics for both modalities were computed as a function of each of the dark/silence times (i.e. inter stimulus intervals). This resulted in 245 illumination conditions, each tested 15.71 ± 0.42 times for each SGN (average ± 95% confidence interval), whereas we did not change the number of acoustical conditions (15 conditions overall).

To simplify the visualization, the average number of spikes per stimulus and the first spike latency jitter were color-coded for SGNs (Fig. 7A,D, optical: *n* = 13 from 2 mice; acoustical: *n* = 26 from 4 mice, CF within the 16 and 32 kHz octave band) and AVCN neurons (Fig. 7B,E, optical: *n* = 16 from 5 mice; acoustic: *n* = 8 from 3 mice, CF within the 16 and 32 kHz octave band). As previously described in this study, the best encoding was achieved at the longest inter-stimulus interval and stimulation strength. The change in the response between SGNs and AVCN neurons was highlighted by computing the difference between the two neuronal populations (Fig. 7C,I). For 38.37% of the optical and 46.67% of the acoustic conditions, the number of spikes per stimulus was increased by at least 0.1 spike/stimulus (i.e. positive neural gain) in the AVCN neurons compared to the SGNs. In contrast, the response decreased by 0.1 in 1.22% of the optical and 6.67% of the acoustic conditions (Fig. 7C,G). The spike jitter decreased for all tested acoustic conditions, and remained similar for most of the optogenetic conditions (Fig. 7F).

Presynaptic terminals of the SGNs converge on the AVCN somas where their glutamatergic inputs trigger post-synaptic action potentials [45,46]. To interpret the neural gain associated with the different spike statistics of the SGNs, it is required to interpret the response from the perspective of the postsynaptic neurons. To do so, population responses were computed across recorded SGNs for all tested illumination conditions (see Methods, Fig. 7G-H,J) and compared to the AVCN neurons’ response of the same condition (Fig. 7I-J). Acoustically, the spike latency jitter across SGNs was similar for all spike rates (Fig. 7J). In contrast, optogenetically, the spike latency jitter across SGNs tended to decrease with the number of spikes per light pulse (Fig. 7J). Strikingly, different illumination conditions were characterized by the same number of spikes per light pulse but different levels of spike synchronization (i.e. the spike latency jitter) within SGNs and thus enabled to individually test the role of the spike synchronization on the entrainment of the response made by AVCN neurons. As illustrated in Fig. 7G-H, the response of AVCN neurons was entrained compared to their inputs when the spike latency jitter within the SGN population was low. Based on the optogenetic response, two regions of interest (ROI) were defined where the number of spikes remained constant but the jitter varied across SGNs (Fig. 7J). For both ROIs, the neural gain in the optogenetic modality was negatively correlated with the jitter (ROI 1: coefficient of correlation, R = −0.733, *P =* 1 × 10^−7^; ROI 2: R = −0.821, P = 3 × 10^−4^), suggesting that synchronous SGN inputs on AVCN neurons are required to increase the rate representation of the stimulus in the latter neurons (Fig. 7K).

## 3 Discussion

Mediated by the ultrafast, trafficking-optimized Chronos, here, we parametrized optogenetic encoding in the firing of SGNs and the recoding of the stimulus by AVCN neurons in mice. We have demonstrated graded optogenetic SGN activation with varying pulse widths, similar to SGN responses to acoustic clicks with different sound pressure levels. We found that the timing of optogenetically-evoked spikes varied little across iterations for a given SGN, but the spike time jitter within a SGN population was similar to what is measured acoustically. Under our experimental condition (maximum light intensity at the optical fiber tip ∼ 35 mW), we identified an effective light pulse width of 1.6 ms to optimally encode the light intensity by achieving the highest maximal discharge rate and the widest dynamic range. Interestingly, the lowest activation threshold in terms of energy was obtained for light pulse trains composed of short light pulses. Upon high rates of stimulation, we observed robust spike rate adaptation that eventually resulted in spike failure. We attributed this to a depolarization block that required a few tens of milliseconds to recover, independently of the tested light intensities. Finally, we implemented a rapid measurement (within minutes) of the input/output function from light to SGN firing by using a semi-stochastic stimulus. The spike statistic, generated by this stimulus, within the SGN population (i.e. the number of spikes and the spike timing) also enabled us to study the recoding of the spike trains by AVCN neurons. We observed that for similar spike rates, synchronized (i.e. low spike latency jitter) excitatory inputs (e.g. the SGN population) on AVCN neurons are associated with entrainment of the AVCN neurons.

### 3.1 Achieving gradual Chronos-mediated activation of the SGNs by changing the pulse width

A dependence of the photocurrent amplitude on the photon flux has already been shown in earlier studies of algal rhodopsins [47] and therefore the manipulation of the light pulse amplitude or its width enables one to control the number of action potentials mediated by those photocurrents (e.g. Refs. [15,16,18,23,48,49]). The application of this concept is key to control laser diodes within their optimal operating range (e.g. best efficiency achieved with short and strong driver currents). In contrast, the application of this concept to gradually activate neural networks, to our knowledge, has not yet been widely employed. In combination with an electrophysiological or optical read-out of the neuronal response, it enables measuring the input/output function between one element of a given network to another. In the context of function restoration, it is of prime importance to establish an appropriate set of stimulation parameters to implement in the coding strategies of future optogenetic prosthetics, such as the optical cochlear implant [3,8]. Both applications require empirical measurements of optogenetically evoked spiking which depends on the opsin's biophysics [15,16,19], the opsin's expression [50] and the neuron excitability [15,17]. Using the auditory system as a model circuit with exquisite temporal fidelity here, we confirmed that gradual activation of SGNs expressing Chronos-ES/TS up to 1.15 spikes/pulse can be achieved by individual light pulses with long durations (i.e. 3.2 ms, Fig. 1E). Interestingly, for pulse trains (100 pps), the number of spikes per pulse peaked at 0.74 for pulses of 1.6 ms and decreased for shorter or longer light pulse durations (Fig. 1J). It was also observed for the longest light pulse that the spike adaptation was the strongest, which stands in contrast with the fact that such adaptation was not observed for acoustically-driven SGNs (Fig. S2.B). This suggests that the dark time between two consecutive pulses might have been too short to enable recovery of Chronos-mediated photocurrents.

### 3.2 Recovery from light-induced masking

Sustained firing could not be recorded from any SGN when stimulated by trains of light pulses at high repetition rates (i.e. 316 and 1000 pps, Fig. S2.C,E). This observation was unexpected given the fast closing kinetics reported for Chronos [14,16]. However, such rapid spike rate adaptation is consistent with our previous recordings of SGN firing mediated by fast opsins (Chronos-ES/TS [14] and vf-Chrimson [13]). Current-clamp recordings of SGNs have revealed that most of the SGNs are characterized by a rapid or intermediate adapting firing in response to current injections [25,51]. Other studies have shown that neurons that were rapidly adapting to a current injection are likely to have an optogenetically induced depolarization block when stimulated with long light pulse durations or high stimulation rates [17,19]. We investigated the time needed to recover from the depolarization block using a so-called forward masking protocol. The masker could induce complete adaptation in approximately 85% of the recorded SGNs, which is similar to the number of rapidly adapting SGNs reported by patch-clamp studies on cultured neonatal SGNs [51]. The best strategy to reach high firing rates of fast-adapting neurons is yet to be elaborated and should likely aim to eliminate the plateau potential (as previously shown with ChETA in fast-spiking neurons [23]. A promising possibility includes the activation of hyperpolarizing opsins such as anion permeating ChRs [52] right after a spike was optogenetically triggered.

The presence of an optogenetically induced masking, which requires tens to hundreds of milliseconds to recover, highlights the importance of empirically determining the appropriate stimulation parameters for applications such as functional restoration and neural circuit analysis. For example, this information is paramount for designing sound coding strategies for future optical cochlear implants, as well as studying the integration of the SGN firing in complex auditory brainstem neural circuits.

### 3.3 Optogenetic encoding of light intensity

Encoding the intensity of a stimulus is critical in the context of sensory restoration. Optically, it corresponds to the number of photons reaching the ChRs, which can be controlled by the pulse width and/or the light intensity. Previously, we examined the range of light intensity associated with a change in the firing rate of the SGNs expressing the red-shifted f- and vf-Chrimson [13]. We reported that the dynamic range for single neurons spanned between 0.67 and 3.77 dB. It seemed to be opsin-dependent but was less than measured acoustically (∼25 dB, [34]) but more than electrically (∼ 1 dB, [39]). Here, we investigated the co-variation of the light intensity and the pulse width to investigate how they jointly impact the intensity encoding. Using 1.6 ms light pulses, we achieved an optimal intensity encoding defined by the lowest intensity threshold, expressed as power (1.50 dB_rel to oABR threshold_), the widest dynamic range (2.26 dB) and the highest driven rate (84.45 spikes/s, Fig. 3). This duration is consistent with the lifetime of ChR2's open state (∼1.5 ms), during which the opsin is integrating photons [53,54]. A longer light pulse could accommodate more than one ChR opening and by that most likely increase the variance and adaptation of the photocurrents.The lowest thresholds, in terms of energy consumption, were measured for the shortest light pulses (0.4 and 0.8 ms, Fig. 3C) and amounted to ∼20 μJ per light pulse. For single SGN expressing Chronos-ES/TS, the energy per pulse is twice bigger than measured per oABR at 10 pps from the same animals, and one order of magnitude bigger than previously reported for f-Chrimson [15] and CatCh [9,21].

### 3.4 Optogenetic encoding of the timing

Reliable optogenetic encoding of the spike timing is critical to resolve neural circuits for which time matters [55], such as the auditory system where spike timing is critical to localize low-frequency sounds in the horizontal plane, discriminate the pitch or detect sound in noise [40,56,57]. Our current and previous works have shown a significantly higher temporal precision of firing for optogenetic than for acoustic stimulation (using clicks) when evaluated at a single neuron level (Fig. 1G,L, [13]). Indeed, this high temporal precision is measured for a given neuron in response to multiple iterations of the same stimulus, which can be interpreted as a highly reliable encoding of the light pulses. Importantly, we report that the latency at which neurons are firing is heterogeneous (Fig. 1F,K, Fig. 2B, Fig. 6G) and therefore, the excitatory inputs of a population of SGNs to the postsynaptic neurons might be less precisely timed than initially estimated. As an attempt to address this question, we computed the precision of the spikes within a population of SGNs built from all the recordings we performed of single SGNs and compared it to acoustically evoked SGNs (Fig. 2D-E). This analysis revealed comparable spike time precisions between the two modalities, which suggests that neurons downstream of SGNs might be excited in a near-physiological manner when optogenetically stimulating SGNs.

### 3.5 The input/output function from light to SGN firing

Empirically measuring the illumination parameters required for an application of optogenetics in neural network analysis or sensory restoration is a prerequisite, as predictions are challenged by the variability of neural excitability, expression levels and biophysical properties of the opsin [15,17,19,23,58]. Estimating those parameters using deterministic stimuli is almost impossible given the limited stability and time scope of recordings of SGN firing (e.g. *in vivo* juxtacellular recordings of the SGN axons). This limitation only allowed a sparse estimation of the input/output function from light stimulation to SGN firing so far [10,[13], [14], [15],^21^]. Instead, using a semi-stochastic stimulus containing many parameters of interest allowed us to measure 35 conditions of different repetition rates and light pulse durations (or 245 conditions of dark intervals and light pulse durations) in ∼200 s in order to estimate the input/output function (Fig. 5). By doing so, we not only replicated the results obtained from the deterministic approach previously used (Fig. 1) but also measured intermediate values. For the first time, the number of conditions per neuron measured as paired data enabled us to identify three distinct clusters of SGNs, which respectively differed in their encoding of the light pulses and their recovery time of the depolarization block (Fig. 6). The neurons requiring less light to fire were the same ones recovering faster from the depolarization block. It potentially suggests a difference in their opsin expression level or neuron excitability. Future studies should address the underlying mechanisms by labeling the recorded SGNs to discriminate between molecular or morphological differences between them [[26], [27], [28],^59^], by examining the difference in their distance to the light emitter, or by investigating the difference of the opsin expression levels [50].

### 3.6 Integration at anteroventral cochlear nucleus neurons

Axons of the SGNs are converging on the somas of different cell types where they provide strong excitatory inputs [45,46,60,61]. Within the AVCN, the bushy cells increase the entrainment and precision of their cycle-to-cycle response to low-frequency sounds [29,30], compared to the SGNs, but the underlying mechanisms of this enhancement are still under debate [[62], [63], [64], [65], [66], [67], [68]]. We took advantage of the heterogeneous SGN firing evoked by all conditions of the semi-stochastic optogenetic stimulation aiming to identify the input/output function from SGN to AVCN neuron firing. Acoustically and optogenetically, we observed an increase in the firing of the AVCN neurons in comparison to the SGNs for some of the tested conditions (Fig. 7A-C) which, however, was associated with no or only little change in their spike precision. Optogenetically, the observed neural gain was correlated to the level of synchronization across SGNs suggesting that coincident inputs were required to increase the discharge rate of the AVCN neurons (Fig. 7G-I), while, in contrast, no correlations were observed acoustically. The precise mechanisms explaining these results are unclear and future studies will require to identify and group AVCN neurons according to their cell types (e.g. spherical bushy cell, globular bushy cell, stellate cells, …), thereby taking into account their morphology and physiology when interpreting the recorded data. Nonetheless, these results support the concept that employing additional stimulation modalities such as optogenetics will help to reveal unknown properties of the elements constituting neural circuits and will contribute to their understanding.

### 3.7 Lessons learned for optogenetic coding

The results of this study inform about efforts for improved functional restoration in diseased sensory and motor systems by using optogenetic neural stimulation. As Chronos is the fastest naturally occurring ChR to date and SGNs serve as targets that demand a very high temporal fidelity of coding, the strong spike rate adaptation, which we observed, indicates an upper bound of stimulation rates for continuous stimulus encoding (∼200 pps, our data and [14]). This likely generalizes to photostimulation mediated by currently known algal ChRs. Recordings of the electrically evoked firing of cat SGNs showed reliable responses to stimulation rates of at least 250 pps [39]. This motivates future efforts to identify or engineer ChRs with large single-channel conductance and fast closing kinetics for more efficient optogenetic SGN stimulation at high rates.

Moreover, high-intensity light pulses with graded effective durations in the range from 400 μs to 2 ms seem appropriate as the “fundamental” stimulus. Considering that operating laser diodes are most efficient with short (μs) and strong (tens of mA) driver currents, this knowledge can be synthesized with our data to develop effective fundamental stimuli from μs pulselets. The *in vivo* data presented here indicate that SGNs readily integrate the photocurrents elicited by such short pulses, which seems in line with previous patch-clamp studies on spike generation [25,44,51,69]. It will be of interest for the ultimate design of optogenetic sound coding strategies to carefully parametrize the effects of pulselet durations and duty cycles of such synthetic stimuli in future experimental and theoretical studies. Likewise, challenging patch-clamp recordings of SGNs expressing ChRs (e.g. [15]) will be needed to elucidate the masking upon optogenetic stimulation. Regardless of the precise underlying mechanisms, the masking places an upper limit on the stimulation rate for reliable sound encoding. Finally, the present study reveals the diversity of optogenetically driven SGN firing that expands the coding capacity by a SGN population well beyond that of an individual SGN. From a theoretical perspective, functional diversity across a population of neurons is a key factor to widen the amount of encoded information and increase its fidelity [70]. The presence of functionally different SGN clusters obtained with the administration of a single viral vector-promotor-ChR combination provides a flavor of what could be obtained when targeting different ChRs to molecularly distinct SGN type I subtypes. While the preparation of a first clinical trial favors to avoid the potential risk which is generated by additional complexity, future generations of optogenetic research for hearing restoration could capitalize on these exciting opportunities.

## 4 Material and methods

### 4.1 Animals

Data were obtained from 73 adult (>12 weeks of age) C57Bl/6 wild-type mice of either sex. For all procedures, animals were placed on a heating pad and body temperature was maintained at 37 °C. All experimental procedures were done in compliance with the German national animal care guidelines and approved by the local animal welfare committee of the University Medical Center Göttingen, as well as the animal welfare office of the state of Lower Saxony, Germany (LAVES).

### 4.2 Virus purification

The virus purification procedure was performed as previously published [14] and an extensive description is available in Ref. [72]. In brief, triple transfection of HEK-293T cells was performed using the pHelper plasmid (TaKaRa, USA), the *trans*-plasmid with PHP.B capsid (Ben Deverman, Viviana Gradinaru, Caltech, USA) and the *cis*-plasmid with Chronos-ES/TS under the control of the human synapsin promotor. Cells were regularly tested for mycoplasma contamination. Viral particles were harvested 72 h after transfection from the medium and 120 h after transfection from the cells and the medium; precipitated with 40% polyethylene glycol 8000 (Acros Organics, Germany) in 500 mM NaCl for 2 h at 4 °C and then after centrifugation combined with cell pellets for processing. Cell pellets were suspended in 500 mM NaCl, 40 mM Tris, 2.5 mM MgCl_2_, pH 8, and 100 U/ml of salt-activated nuclease (Arcticzymes, USA) at 37 °C for 30 min. Afterward, the cell lysates were clarified by centrifugation at 2000×*g* for 10 min and then purified over iodixanol (Optiprep, Axis Shield, Norway) step gradients (15%, 25%, 40%, and 60%) at 350,000×*g* for 2.25 h. Viruses were concentrated using Amicon filters (EMD, UFC910024) and formulated in sterile phosphate-buffered saline (PBS) supplemented with 0.001% Pluronic F-68 (Gibco, Germany). Virus titers were measured using a AAV titration kit (TaKaRa/Clontech) according to the manufacturer's instructions by determining the number of DNase I resistant vg using qPCR (StepOne, Applied Biosystems). The purity of produced viruses was routinely checked by silver stainings (Pierce, Germany) after gel electrophoresis (Novex™ 4–12% Tris–Glycine, Thermo Fisher Scientific) according to the manufacturer's instruction. The presence of viral capsid proteins was positively confirmed in all virus preparations. Viral stocks were kept at −80 °C until injection.

### 4.3 Virus injection

Virus injection was performed as previously published [14] and an extensive description is available [72]. In brief, PHP.B vectors carrying the targeting-optimized Chronos (Chronos-ES/TS) linked to the reporter-protein eYFP and under control of the human synapsin promotor (titer: 3.3 - 8.4 × 10^12^ genome copies/ml) was injected in the left ear at P5–P7. Under general isoflurane anesthesia and analgesia achieved through local application of xylocaine and subcutaneous injection of burprenorphine (0.1 mg/kg) and carprofen (5 mg/kg), the left bulla was approached via a dorsal incision. Injection of 1–1.5 μl of virus suspension (mixed with fast green, 1:20) was performed in the scale tympani using laser-pulled (P-2000, Sutter Instrument Inc., USA) quartz capillaries (Science Products, Germany) connected to a pressure micro-injector (100–125 PSI, PLI-100 picoinjector, Harvard Apparatus). After the virus application, the tissue above the injection site was repositioned and the wound was sutured. Recovery of the animals was then daily tracked for the first week and weekly tracked until the electrophysiological recordings. If needed, additional application of carprofen (5 mg/kg) could be performed. Animals were kept in a 12-h light/dark cycle, with access to food and water *ad libitum*.

### 4.4 Stimulation

Stimuli were generated via a custom-made system based on NI-DAQ-Cards (NI PCI-6229; National Instruments; Austin, USA) controlled with custom-written MATLAB scripts (The MathWorks, Inc.; Natick, USA). Acoustic stimuli (sampling rate = 830 kHz) were presented open-field via a loudspeaker (Avisoft Inc., Germany) localized on the left side at ∼15 cm from the pinna. A 0.25-inch microphone and measurement amplifier (D4039; 2610; Brüel & Kjaer GmbH, Naerum, Denmark) were used to calibrate sound pressure levels. Optical stimuli were delivered at the cochlear round window via an optical fiber (200 μm diameter, 0.39 NA, Thorlabs GmbH, Germany) coupled to a blue laser (473 nm, MLLFN-473-100, 100 mW diode pumped solid state [DPSS]; Changchun New Industry Optoelectronics). The maximum light intensity (or radiant flux) was measured before every experiment (LaserCheck; Coherent Inc.) and later used for calibration. The round window was exposed as previously described [[13], [14], [15],^72^]. Briefly, the round window was exposed by opening the temporal bone ventrally from the *stylomastoid foramen* and the exact location of the round window was determined by visually following the stapedial artery.

### 4.5 Auditory brainstem recordings (ABR)

Animals were anesthetized via intraperitoneal injection of a mixture of xylazine (5 mg/kg) and urethane (1.32 mg/kg) and appropriate analgesia was achieved by sub-cutaneous injection of buprenorphine (0.1 mg/kg, repeated every 4 h) and carprofen (5 mg/kg). Depth of anesthesia was monitored regularly by the absence of reflexes (hind limb withdrawal) and adjusted accordingly. ABRs were recorded using sub-dermal needle electrodes inserted underneath the pinna, on the vertex, on the back near the contralateral leg and signals were amplified using a custom-made differential amplifier, sampled at a rate of 50 kHz, filtered (second order Butterworth filter, 300–3000 Hz) and averaged across 200–1000 iterations. The first ABR wave, reflecting the synchronous activation of the SGNs, was detected semi-automatically with a custom-written MATLAB script. Thresholds were visually defined as the lowest intensity that elicited a reproducible response in the recorded traces.

### 4.6 Juxtacellular recordings of SGNs and AVCN neurons

If positive acoustic or optogenetic ABRs were recorded, the mice were prepared for subsequent juxtacellular recordings of the SGN and AVCN neurons. Briefly, a tracheotomy and intubation were performed before mounting the animal in a custom-designed stereotactic head holder. Pinnae were removed, the scalp reflected, portions of the lateral interparietal and the left occipital bone removed, and a partial cerebellar aspiration was performed to expose the left semi-circular canal. A reference electrode was placed on the contralateral muscles behind the right ear. A borosilicate sharp micropipette (∼50 MΩ, 3 M NaCl) was navigated from a reference point at the surface of the semi-circular canal to a stereotaxic position from where SGNs and AVCN neurons recordings could be obtained (LN Mini 55 micro-manipulator, Luigs & Neumann, Germany). Using the step function from the micromanipulator (3 mm/s, 1.5 μm steps), juxtacellular recordings from SGNs or AVCN neurons were obtained. At the end of the procedure, the relative position of the auditory meatus was visually controlled by exposing it. Signals were amplified (ELC-03XS, NPI electronic, Germany) and digitized at a sampling rate of 50 kHz by the same NI-DAQ-Cards and custom-written MATLAB scripts used for stimulus generation. Spikes were detected offline based on their threshold (manually determined) using custom-made MATLAB scripts.

Differentiation between SGN and AVCN neurons was performed on the sole basis of 3 criteria. Units were classified as SGNs if their depth relative to the cerebellum surface ≥1000 μm, their first spike latency ≤3 ms in response to 0.8–1.6 ms light pulse presented at maximum (light intensity/80 dB SPL_PE_ acoustic click, and if their action potentials were monophasic and positive.

### 4.7 Stimulation strength

The stimulation strength was defined acoustically as the sound pressure level (SPL click intensity) in dB SPL_PE_ (PE: peak equivalent, [[42], [73]]) and optogenetically as the pulse duration. Previous measures have shown that the oABR amplitude increases with the pulse duration, thus suggesting an increased firing synchronization between SGNs (Fig. S1.C, [14]) with that parameter. Evaluation of the stimulus strength was either achieved by single stimuli (presented at 10 pps, 80 presentations) or stimulus trains (100–1000 pps, 20 iterations, 400 ms light/sound, 100 ms dark/silence). To optimize acquisition time and enable comparisons between all pulse duration of interests, 2 s stimulation containing 4 blocks of 500 ms were built (400 ms). Acoustically, acquisitions of the four different repetitions rates (10, 100, 316 and 1000 cps) were performed. Following successful acquisition at 80 dB SPL_PE_, acquisitions were repeated at either 60 and 70 dB SPL_PE_ in a random manner. Only units acquired at the three sound intensities were included. Optogenetically, acquisitions of four different light pulse durations (0.4, 0.8, 1.6 and 3.2 ms) were performed at either 10, 100, 316 or 1000 pps (if the pulse duration exceeded the stimulation period, no acquisition was done for that condition).

The number of spikes evoked per stimulus was counted from the 10 ms following each stimulus presentation. The first spike following each stimulus was detected and its average latency and latency jitter (i.e. standard deviation of the latency) were computed. The adaptation ratio was computed as the ratio between the discharge rate during the first 50 ms of the train and the whole duration of the stimulation train.

### 4.8 Population response

To estimate the representation of the stimulus across a population of SGNs, we built neurograms from all recorded SGNs in response to the given stimulus. Raster plots were built from all recorded SGNs by randomly selecting one iteration per SGN. The number of spikes per stimulus, first spike latency and first spike latency jitter across SGNs were computed and the operation was repeated 50 times (e.g. bootstraps).

### 4.9 Intensity

Intensity analysis was performed as paired data by varying the light pulse duration (0.4, 0.8, 1.6 and 3.2 ms) and the light intensity of a given stimulus at 100 pps which was presented as already mentioned above. The stimulus amplitude was related to the threshold of the auditory brainstem responses (see Ref. [13]). Light intensity was expressed in dB_oABR threshold (mW)_ = 10 × log10 (A/A0) where A is the presented light intensity and A0 the light intensity at oABR threshold. Stimuli were presented first at maximum laser output and repeated at as many other intensities as the unit could be held to cover the full dynamic range (i.e. from threshold to saturation). Only units for which at least 4 intensities were measured were included. We previously showed that SGN firing at the threshold is characterized by a strong adaptation [13], therefore rate-level functions (RLF) and subsequent analysis were only computed from the plateau response (between 100 and 400 ms). The spontaneous activity of each neuron was measured from the lowest sub-threshold intensity at 0.4 ms and RLFs were classified as responding if their discharge rate at the highest intensity was 10 spikes/s above the spontaneous discharge rate. Responding RLFs were classified as saturating if the slope measured between the discharge rate at the two highest measured intensities was ≤3 spikes/dB, else they were classified as non-saturating. Per RLF, if sub-threshold intensities were measured, we computed an interpolated d-prime-level function (with the spontaneous activity as a reference, linear interpolation with a 0.2 dB step) from which we defined the threshold as the lowest intensity reaching a d-prime ≥1 [13,40,41]. Saturated RLFs were fitted with a sigmoidal equation and the dynamic range (i.e. the difference in intensity) and the light-driven rate (i.e. the difference in discharge rate) were measured between the threshold and the intensity leading to 95% of the maximum discharge rate. Dynamic range and light-driven rate from unsaturated RLFs were measured between the threshold and the maximum tested intensity.

### 4.10 Forward masking protocol

Recovery from the optogenetically induced firing refractoriness was measured by a so-called forward masking protocol. Ten light pulses of 1.6 ms presented at 313 pps were used as a masker to induce firing adaptation and the recovery was measured at different time intervals by a single light pulse (i.e. the probe) of 1.6 ms. A total of 20-time intervals ranging between 4 and 180 ms were tested and a 200 ms dark time was applied between each measure. Each interval was tested 20 times. Stimuli were presented first at maximum laser output and repeated at as many other intensities as the unit could be held. The success of the masker to fully adapt the firing was evaluated from the discharge rate and the adaptation ratio of the masker. Next, the spike probability (i.e. the probability of the probe to evoke spikes) per interval was measured and normalized to the spike probability in response to the longest interval. Recovery curves were fitted by a mathematical model ($y=\max\left(0,a\times e^{\frac{-\left(k+x\right)}{b}}\right)$ to define the time to absolute recovery (i.e. time required to recover any firing, - k) and relative recovery (i.e. time required to recover 95% of the spike probability, - k + (3 x b)). Additionally, the spontaneous discharge rate of the SGNs was measured in the last 180 ms of the 200 ms dark time following each probe.

### 4.11 Semi-stochastic stimulus

A semi-stochastic stimulus was designed for simultaneous testing of 35 combinations of repetition rates (10, 56, 100, 179 and 313 pps) and light pulse durations (0.2, 0.4, 0.6, 0.8, 1.2, 1.6, 2.4 ms). Each combination was randomly tested 10 times per stimulus iteration and the presentation order was randomized for each of the 20 iterations, resulting in 200 presentations per combination. Each iteration started with a masker composed of 10 light pulses of 2.4 ms presented at 100 pps and finished with 200 ms of dark. Additionally, a forward masking protocol testing for the intervals of 15 and 80 ms was included. Recordings were made at maximum light intensity. Similarly, an acoustic stimulus was designed using acoustic click (100 μs) testing for 5 repetition rates (10, 56, 100, 179 and 313 cps). Recordings were made at 60, 70 and 80 dB SPL_PE_. Raster plots were built from a 3 ms window following each stimulus which was defined by first computing a spike-triggered average. For each condition, the number of spikes per stimulus, first spike latency and first spike latency were computed as described above. We determined, using a classifier, that at least 12 iterations of the stimulus (120 repetitions per condition) were needed to reliably estimate the response (Fig. S5).

### 4.12 Cochlear histology

Following each optogenetic injection, cochleae from both side were prepared as previously described [[13], [14], [15]]. Briefly, samples were fixed for an hour in 4% paraformaldehyde (PFA), decalcified for 3–4 days in 0.12 M ethylenediaminetetraacetic acid (EDTA), dehydrated in 25% sucrose solution for 24 h and cryosectioned (mid-modiolar cryosections, 25 μm tick). Immunofluorescence stainings were done in 16% goat serum dilution buffer (16% normal goat serum, 450 mM NaCl, 0.6% Triton X-100, 20 mM phosphate buffer, pH 7.4). The following primary antibodies were used at 4 °C overnight: chicken anti-GFP (1:500, ab13970 Abcam, USA) and guinea pig anti-parvalbumin (1:300, 195004 Synaptic Systems, Germany); and secondary antibodies for 1 h in room temperature: goat anti-chicken 488 IgG (1:200, A-11039 Thermo Fisher Scientific, USA) and goat anti-guinea pig 568 IgG (1:200, A-1107 Thermo Fisher Scientific, USA). Finally, slices were mounted in Mowiol 4–88 (Carl Roth, Germany). Samples were imaged with a LSM 510 Zeiss Confocal Microscope (Zeiss, Germany) mounted with a 40x air objective. An image per cochlear turn (apex, middle and base) was taken from the modiolus (i.e. the bony structure containing the SGN somas). Image analysis was performed by a custom-written MATLAB script modified from Ref. [71]. Briefly, SGN somas and modiolus areas were manually detected using a touch screen from the parvalbumin images. Next, individual somas were automatically segmented method from every Z-stack using Otsu's threshold and a mask corresponding to the given SGN was defined for the Z-stack for which the mask was fulfilling the criteria of size (area and diameter) and circularity. In case the segmentation was not right, the segmentation of the given SGN soma was performed manually. Next, the median GFP brightness of each SGNs was measured and its distribution was fitted with a Gaussian mixture model with up to three components. A threshold, above which SGNs somas were considered as transduced, was defined as average + 3 x standard deviation of the Gaussian distribution with the lowest mean.

### 4.13 Analysis

Data were analyzed using Matlab (MathWorks). All averages in text and figures were expressed as mean ± 95% confidence interval. Normality was tested by a Jarque-Bera test. For statistical comparison between 2 independent groups, a two-sample *t*-test (parametric) or a Wilcoxon rank sum test (non-parametric) were used. For statistical comparison between more than 2 independent groups, ANOVA (parametric) or a Kruskal-Wallis test (non-parametric) were used followed by a Tukey's Honest Significant Difference procedure. For statistical comparison between paired data, a Wilcoxon signed rank test on paired samples followed by a Bonferroni correction of the p-values were used.

## Ethics approval

All experimental procedures were done in compliance with the German national animal care guidelines and approved by the local animal welfare committee of the University Medical Center Göttingen, as well as the animal welfare office of the state of Lower Saxony, Germany (LAVES).

## Consent for publication

Not applicable.

## Availability of data and materials

Data and materials are available upon reasonable request to the authors.

## Funding

This work was funded by the 10.13039/501100000781European Research Council through the Advanced Grant ‘OptoHear” to T.M. under the European Union's Horizon 2020 Research and Innovation program (grant agreement No. 670759), the Fraunhofer and Max-Planck cooperation program (NeurOpto grant) to T.M., the German Research Foundation through the Priority Program 1926 “Next generation optogenetics” to A.H. and T.M. and was funded by the Deutsche Forschungsgemeinschaft (DFG, German Research Foundation) under Germany’s Excellence Strategy - EXC 2067/1- 390729940 to T.M. and A.H., as well as the Leibniz Program to T.M. A.M. is recipient of a scholarship of the Göttingen Promotionskolleg für Medizinstudierende, funded by the Jacob-Henle-Programm or Else-Kröner-Fresenius-Stiftung (Promotionskolleg für Epigenomik und Genomdynamik, 2017_Promotionskolleg.04). In addition, this research was supported by Fondation Pour l’Audition (FPA RD-2020-10) to TM.

## Credit authorship contribution statement

**Artur Mittring:** Conceptualization, Validation, Formal analysis, Investigation, Data curation, Writing – original draft, Writing – review & editing, Visualization. **Tobias Moser:** Conceptualization, Validation, Resources, Writing – original draft, Writing – review & editing, Supervision, Project administration, Funding acquisition. **Antoine Tarquin Huet:** Conceptualization, Software, Validation, Formal analysis, Investigation, Resources, Data curation, Writing – original draft, Writing – review & editing, Visualization, Supervision, Project administration, Funding acquisition.

## Declaration of competing interest

T.M. is co-founder of Optogentech. The other authors do not have competing interests.
