# Supplementary material for: Graded optogenetic activation of the auditory pathway for hearing restoration
Source: Brain Stimul. 2023 Mar-Apr;16(2):466–83. doi: 10.1016/j.brs.2023.01.1671 (PMC10159867; doi:10.1016/j.brs.2023.01.1671)
Supplement: Multimedia component 1 [file mmc1.docx]

**
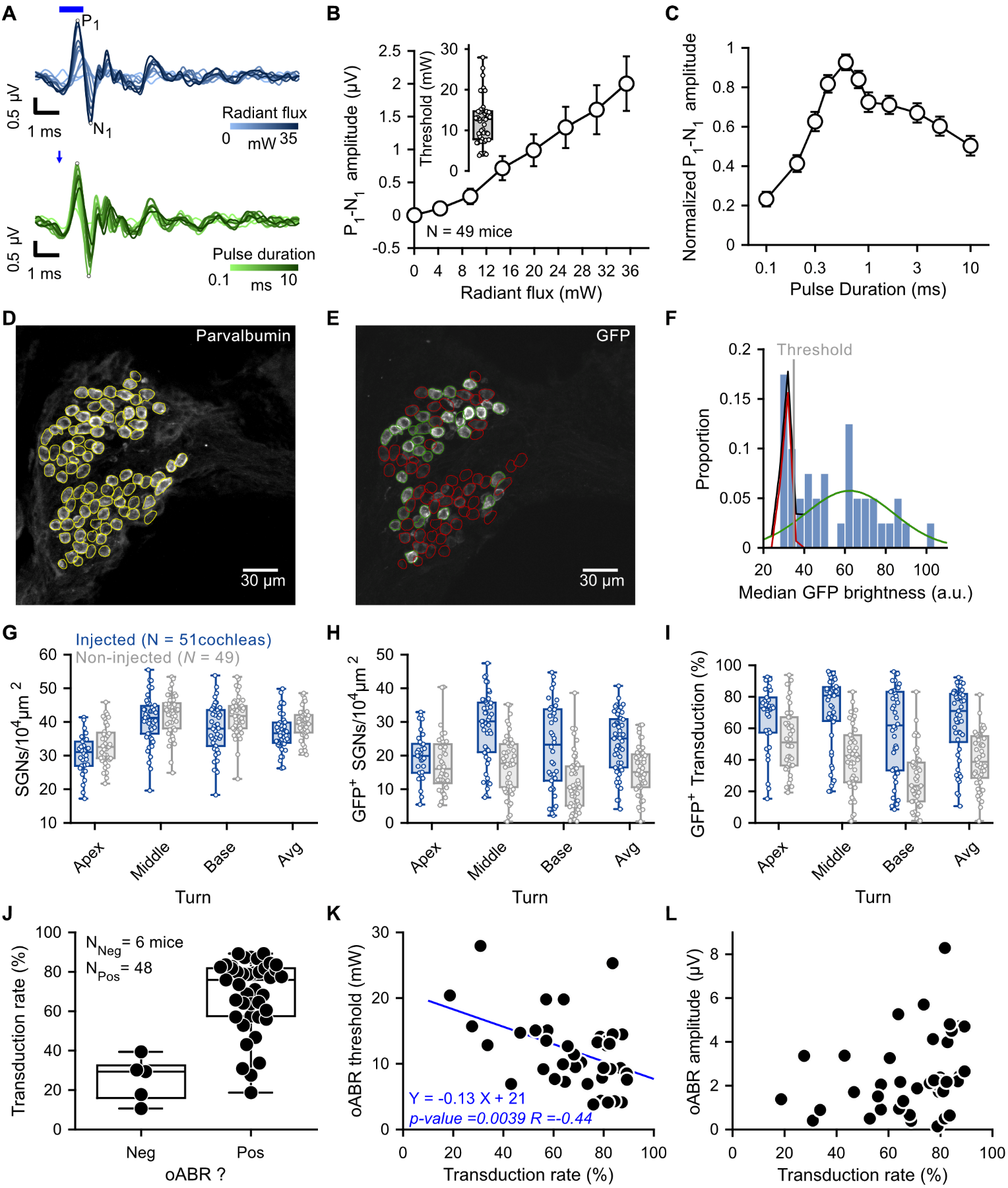
**

**Figure S1: Physiological and histological characterization of the optogenetic modification of murine spiral ganglion neurons using Chronos-ES/TS. A.** Representative optogenetically evoked auditory brainstem responses (oABR) to various light intensities (top panel, blue, 1 ms light pulses presented at 10 pps, the thick blue line represents the light pulse) and light pulse durations (lower panel, green, 35 mW light pulses presented at 10 pps, the blue arrow represents the stimulus onset). The first wave complex (P_1_-N_1_) corresponds to the synchronous activation of the SGNs. **B-C.** Quantification of P_1_-N_1_ amplitude (*N* = 49 mice) as a function of the light intensity (B, light intensities binned per 5 mW steps) and pulse duration (**C.**). Average ± 5% confidence interval. Inset in B.: Box-and-whisker plot (minimum, 25^th^, median, 75^th^ percentile, maximum) of oABR thresholds. **D-F.** Chronos-ES/TS transduction was semi-automatically evaluated from confocal images of mid-modiolar cryosection stained for Parvalbumin (D) and GFP (E). The method was previously described in (71) and segmented individual SGN somas from the Parvalbumin images, measured the median GFP signal from individually segmented SGNs and defined, using a Gaussian-mixture model, the threshold (vertical grey line) at which the median GFP brightness exceeded (average + 3 standard deviations, green curve) the distribution of the GFP background noise (F, red curve). **G-I.** Quantification of the SGN density (i.e number of parvalbumin-positive SGN somas per cross-sectional area of the Rosenthal’s canal, G), the density of GFP positive SGNs (H), and the transduction rate (i.e. the ratio between the GFP positive SGN density and the SGN density, I) for all cochlear turns of injected (blue, *N* = 51 cochleae) and contralateral non-injected (grey, *N* = 49) mouse cochleae. Box plots show minimum, 25th percentile, median, 75th percentile, and maximum with individual data points overlaid. **J.** Quantification of the transduction rate as a function of the oABR result. **K-L.** Correlation of the transduction rate with the oABR threshold (K) and oABR N_1_-P_1_ amplitude (L). In case a significant correlation coefficient (R) was measured, a linear model (blue line) was fitted to the data.


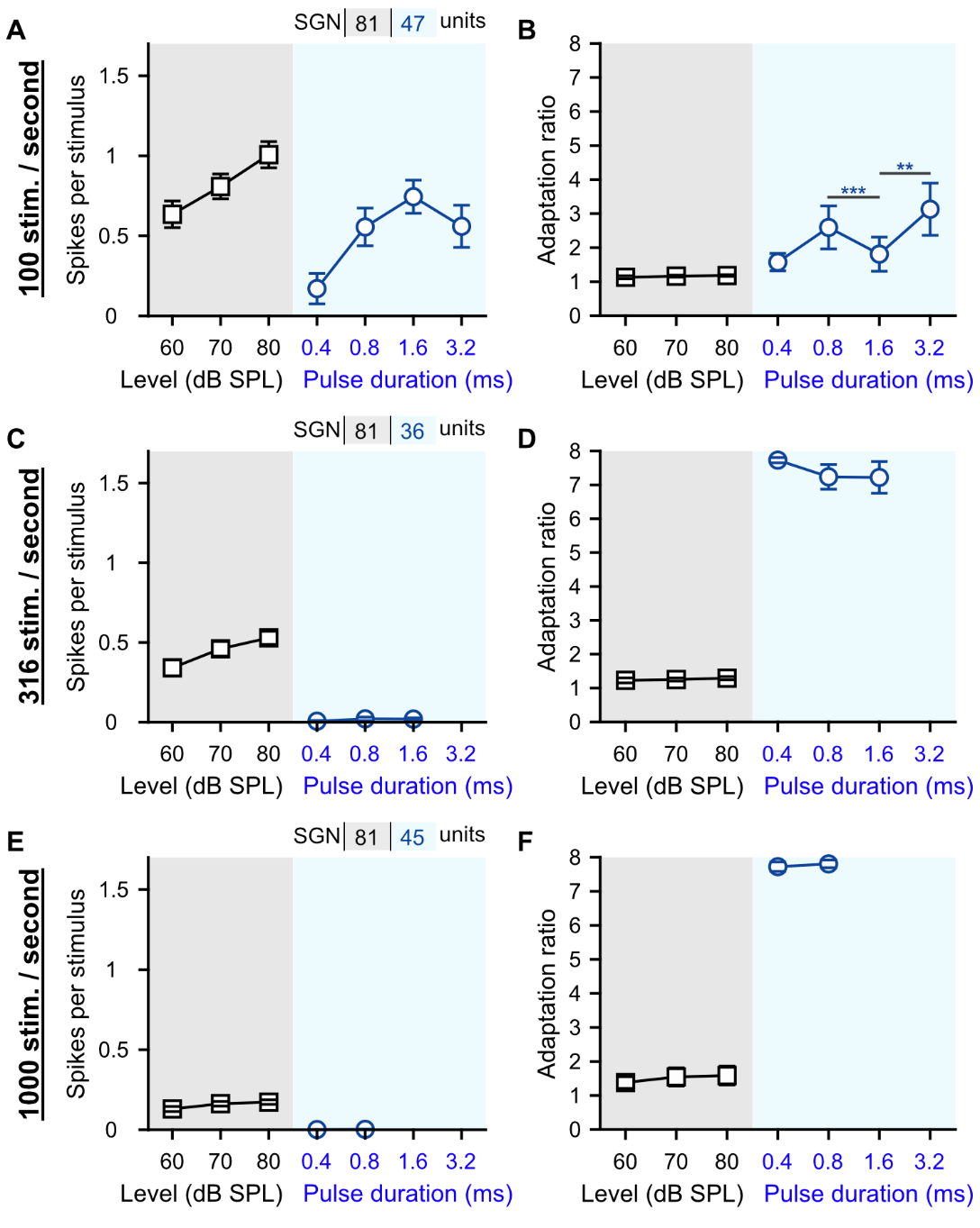


**Figure S2: 316 and 1000 stimulation per second light pulse trains fully adapted the SGN firing.** Quantification of the number of spikes per stimulus (**A, C and E**) and adaptation ratio (i.e. the ratio of the discharge rate during the first 50 ms and the first 400 ms of the stimulation, **B, D and F**) at 100 (**A-B**), 316 (**C-D**) and 1000 stim. per second (**E-F**). The effect of the acoustic levels and light pulse durations was tested by a Wilcoxon signed rank test on paired samples followed by a Bonferroni correction of the *p*-values (**, *P* ≤ .01; ***, *P* ≤ .001).


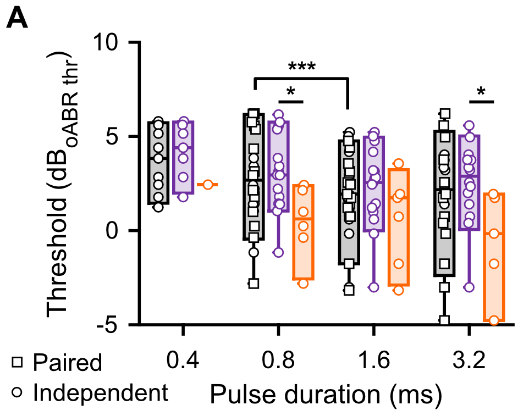


**Figure S3: Quantification of the light intensity threshold as a function of the light pulse duration.** The color code is similar to in figure 3.B. Box plots show minimum, 25th percentile, median, 75th percentile, and maximum with individual data points overlaid. Circles represent independent data points and squares paired data points at 0.8, 1.6 and 3.2 ms. The effect of light pulse durations was tested at 0.8, 1.6 and 3.2 ms using a Wilcoxon signed rank test on paired samples followed by a Bonferroni correction. Following assessment for normality using a Jarque-Bera test, the difference between non-saturated and saturated units was tested accordingly by one-way analysis of variance or a Kruskal-Wallis test (*, *P* ≤ .05; ***, *P* ≤ .001).


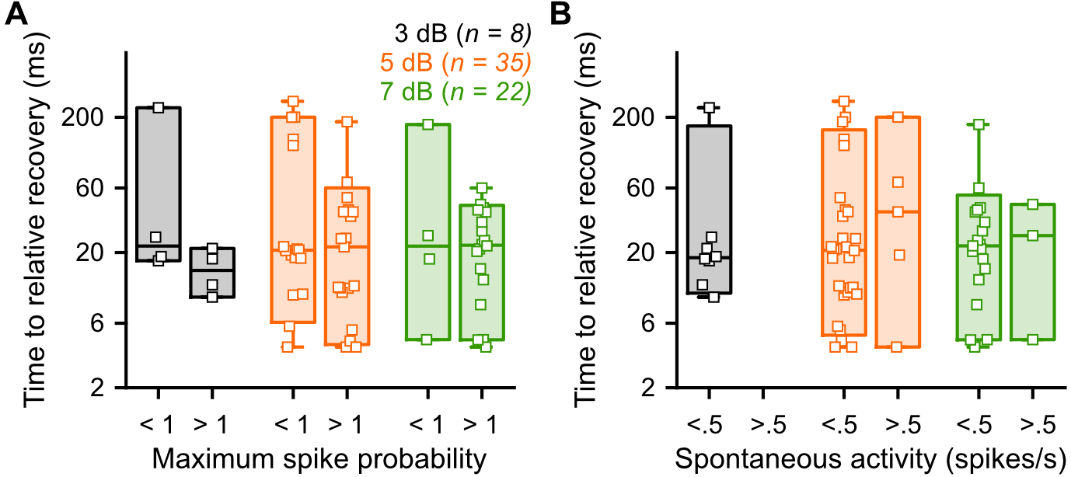


**Figure S4: Time to relative recovery is independent of the spontaneous activity (A) and maximum spike probability (B).** The spontaneous activity was measured in the last 180 ms of the 200 ms of dark following the longest tested interval (Δ t). The maximum spike probability was the spike probability measured in response to the probe eliciting the highest spike probability. Light levels were expressed as dB_relative to oABR threshold_ (bin width = 2 dB) and color-coded.


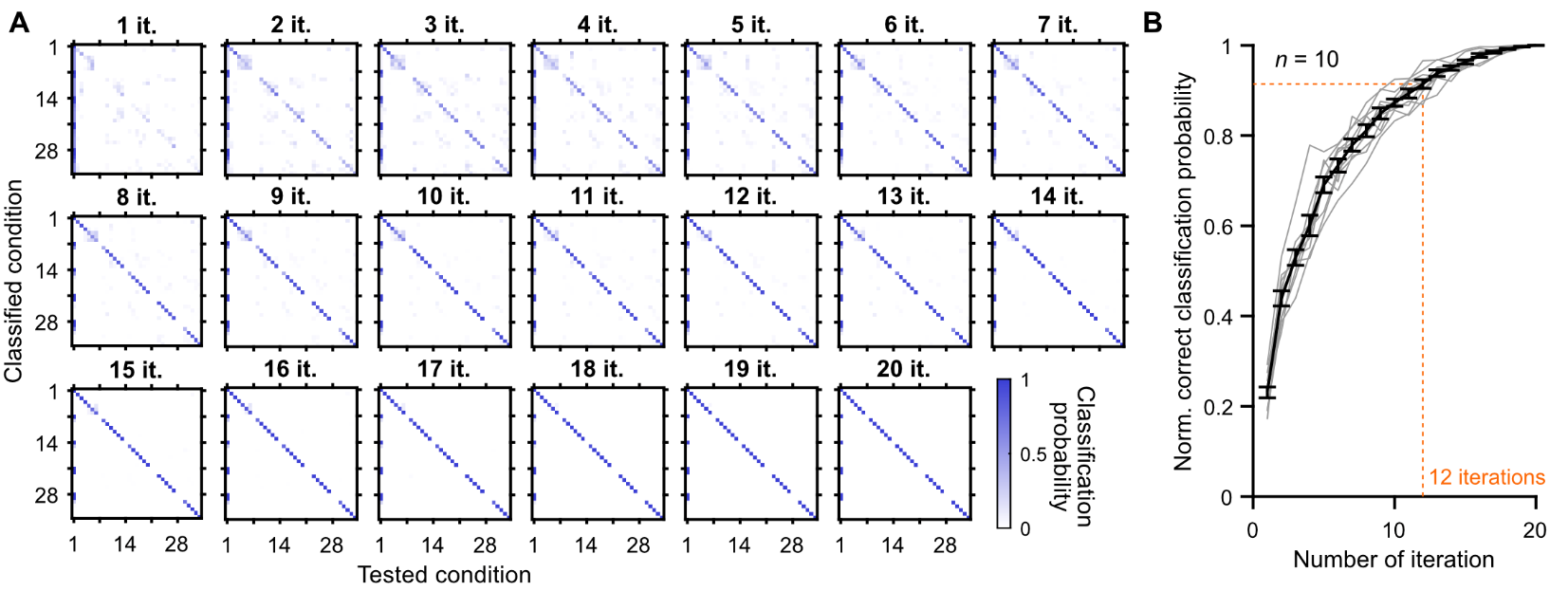
**Figure S5: 12 iterations are needed per condition for the semi-stochastic stimulus.** Subplots are showing the classification probability of 35 tested (based on PSTH, bin width = 50 µs) and classified conditions (7 pulse durations x 5 repetition rates in the semi-stochastic stimulus) for their repetitive presentations (10-200). The plot shows the normalized correct classification probability as a function of the number of presentations. On average 12 iterations are needed to have a correct classification probability of 1.


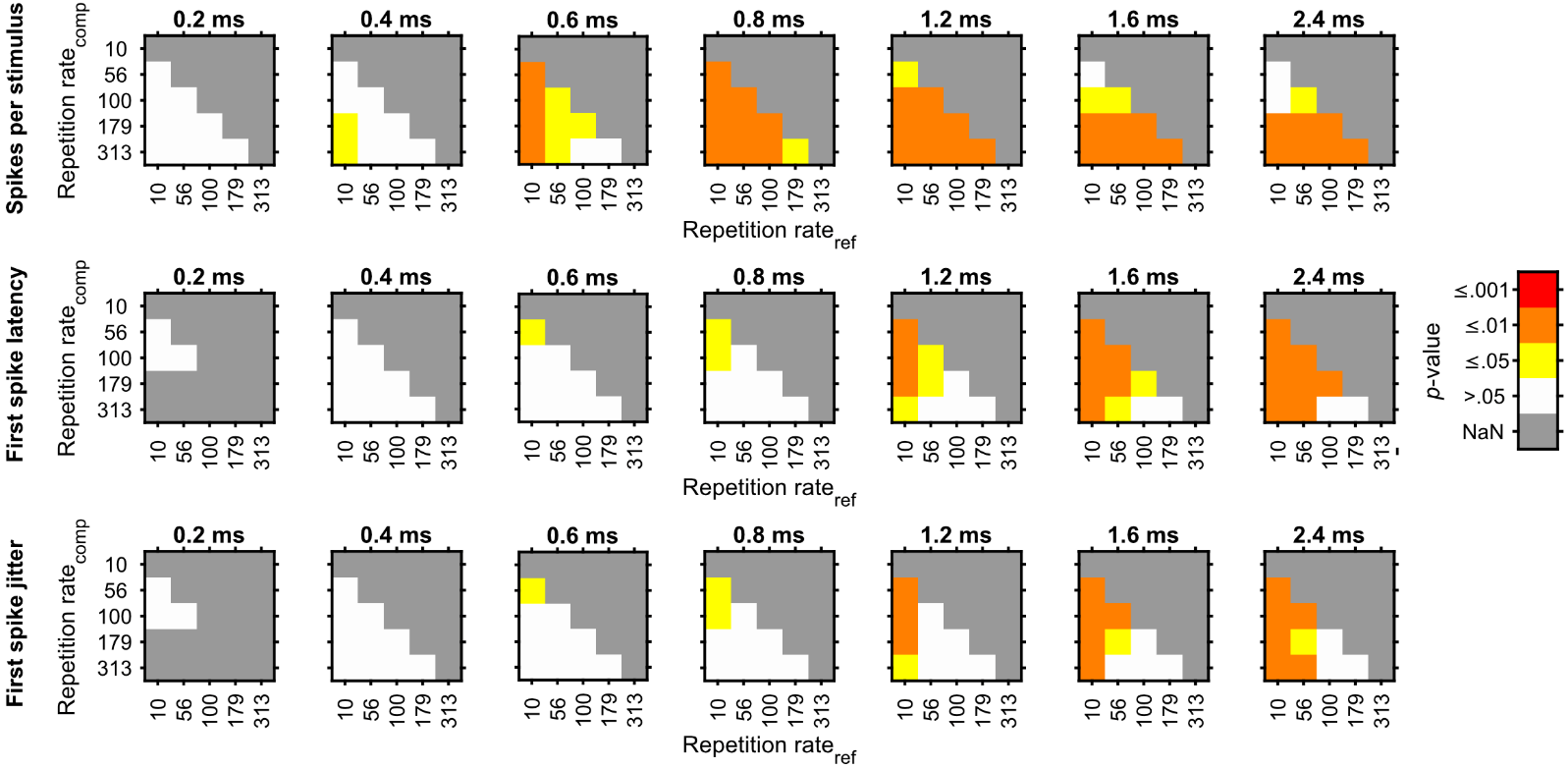


**Figure S6: Results of the hypothesis tests performed on the data from figure 5.E-F. to evaluate the effect of the repetition rate at various pulse durations on the number of spikes per stimulus, first spike latency and first latency spike jitter.** Wilcoxon signed rank test on paired samples followed by a Bonferroni correction test. A color code was used to represent the *p*-values.
